# Millets, dogs, pigs and permanent settlement: productivity transitions in Neolithic northern China

**DOI:** 10.1017/ehs.2024.31

**Published:** 2024-11-11

**Authors:** Chris J. Stevens, Yijie Zhuang, Dorian Q. Fuller

**Affiliations:** 1 UCL Institute of Archaeology, University College London, London WC1H 0PY, UK; 2 School of Archaeology and Museology, Peking University, Peking, China

**Keywords:** Domestication, origins of agriculture, East Asia, Yangshao, Xinglongwa, pigs, millet

## Abstract

The transition to sedentary agricultural societies in northern China fuelled considerable demographic growth from 5000 to 2000 BC. In this article, we draw together archaeobotanical, zooarchaeological and bioarchaeological data and explore the relationship between several aspects of this transition, with an emphasis on the millet-farming productivity during the Yangshao period and how it facilitated changes in animal husbandry and consolidation of sedentism. We place the period of domestication (the evolution of non-shattering, initial grain size increase and panicle development) between 8300 and 4300 BC. The domestication and post-domestication of foxtail (*Setaria italica*) and broomcorn (*Panicum miliaceum*) millet increased their productivity substantially, with much greater rate of change than for rice (*Oryza sativa*). However, millets are significantly less productive per hectare than wet rice farming, a point reflected in the greater geographical expanse of northern Neolithic millet cultures (5000–3000 BC) in comparison with their Yangtze rice-growing counterparts. The domestication of pigs in the Yellow River region is evidenced by changes in their morphology after 6000 BC, and a transition to a millet-based diet c. 4500–3500 BC. Genetic data and isotopic data from dogs indicate a similar dietary transition from 6000 to 4000 BC, leading to new starch-consuming dog breeds. Significant population increase associated with agricultural transitions arose predominately from the improvement of these crops and animals following domestication, leading to the formation of the first proto-urban centres and the demic-diffusion of millet agriculture beyond central northern China between 4300–2000 BC.

## Graphical abstract

**Figure:**
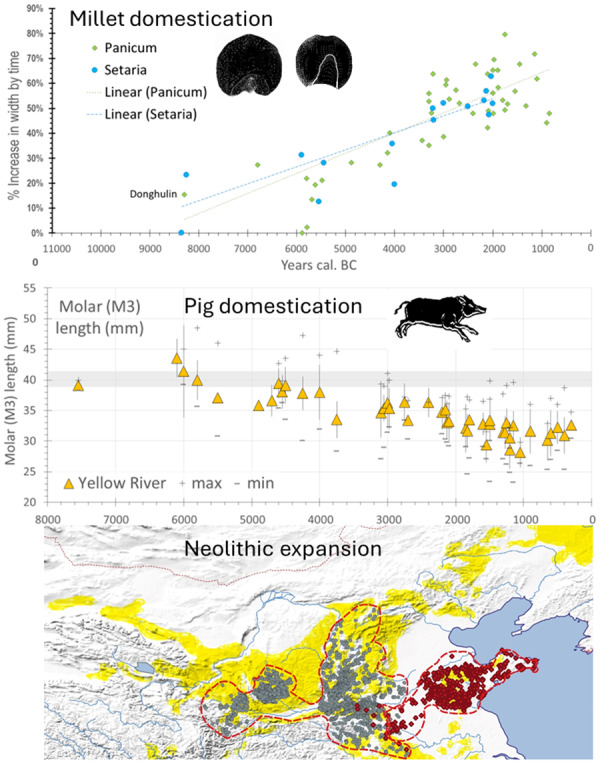

## Introduction

Agricultural origins had profound consequences on long-term human demography and global environments (e.g. Bellwood, 2005; Ellis, 2015). The ability to feed more people from stored crops, usually correlated with sedentism, and the potential to use starch-rich crop plants as early weening foods, worked together to sustain population growth (e.g. Smith, 1976; Harris, 1978; Cohen, 1989). This growth in turn fuelled what has been dubbed the Neolithic Demographic Transition (Bocquet-Appel, 2011), and potentially provided a major motor behind the expansion and migration of Neolithic farmers, carrying their genes and languages (Bellwood, 2005). Nevertheless, the details of the process of this agricultural transition have received less attention in the literature. As recent empirical evidence has made clear, crop domestication processes were usually protracted over millennia (Fuller et al., 2009, 2014; Allaby, 2010; Allaby et al., 2017), and so too were the shifts to reliance on agricultural produce over wild foods (e.g. Fuller and Qin, 2010; Fuller et al., 2018; Arranz-Otaegui et al., 2016; Wallace et al., 2019; Bestel et al. 2018). This was a reliance that for some regions continued through subsequent agricultural expansions (Stevens et al., 2022). This raises a very acute question about when the demographic change that fuelled population growth and expansion of the sort that are inferred from the spread of several major language families occurred. While such a demographic event can be broadly placed within the transition from hunter–gatherer economies to sedentary farming economies, whether such a demographic transition occurred earlier or later in the process still needs to be addressed. In order to tackle this issue, we need multiple lines of evidence relating to the advent of sedentism, and increased starch in the diet, as well as evidence for the transition to cultivation and agriculture based on domesticated species. In terms of considering the expansion of the Neolithic population resulting from demographic increase it is then necessary to have some understanding of productivity and carrying capacity of local communities and their land.

The present paper explores the Neolithic transition in northern China through a consideration of millet productivity and millet domestication alongside proxies from other domestic taxa, namely dogs and pigs that provide additional lines of evidence for the transition to a millet-focused diet and sedentism. We draw together an assessment of how many times these taxa are likely to have been domesticated and brought together as part of the northern Chinese Neolithic system. These four taxa (two millets, dogs and pigs) represent part of an inter-related cultural and economic system that tracks population growth and dispersal across northern China, and provides fundamental background to understanding the long-term distribution of cultural features, including language, and to the subsequent rise of urban civilisation in this region.

## Millet agriculture, productivity and demic-diffusion

The underlying assumption behind Neolithic population expansion is the process of demic diffusion, a process by which growing agricultural communities spread outwards, as populations increase and expand beyond more populated areas (Ammerman & Cavalli-Sfroza, 1971), and pressure on available resources increases. Estimates of rates of demic-diffusion suggest a relatively slow continuous process of expansion in which agricultural communities spread outwards at around 25 km per generation (Stevens et al., 2022). The exact social and demographic mechanisms that drove the diffusion and expansion of agricultural communities across different landscapes often remain ill defined. One variant of demic-diffusion is a more punctuated process of population division referred to as community fission; where a resident population becomes too large to be readily sustained by the existing available land, economic and social systems, so that the population hives off a sub-group of migrants that move outwards in search of new land to settle and farm (see Bellwood, 2005). Rindos (1980) offered the explanation that such ‘emigration events’ will occur when local populations grow to or beyond the immediate carrying capacity of the local environment. In other words, ‘excess’ population is controlled through outwards expansion, or the separation of part of a community to set up a new community on land previously unoccupied by agriculturalists. The concept of carrying capacity is not without its problems (e.g. Harris, 1978; Hassan, 1981: 164). Sahlins’ (1972) study of *Stone Age Economics* concluded that most small-scale societies (hunter–gatherer or farmer) operate well below carrying capacity, in a state of ‘underproduction’. Using data from a range of traditional production systems, their populations and computed potential productive capacity, Sahlins indicates that they all appear to under-produce with only a couple of instances producing at 65 or 75% of capacity, with an average being around 45% of estimated capacity (Sahlins, 1972: 42–48; see also Carlstein, 1980: 239; Dewer, 1984). A recent synthesis of Neolithic evidence from Europe identified a tendency for dispersal to occur when regional populations were growing rapidly but before they reached maximum size (Shennan, 2018). Thus carrying capacity may be less an absolute ecological threshold and more a product of cultural perception and interpretation of environmental circumstances. Nevertheless, the implications are the same: populations will grow towards a limit, and as a population approaches or exceeds that limit, a fission or an outward expansion will occur.

**Figure 1.**
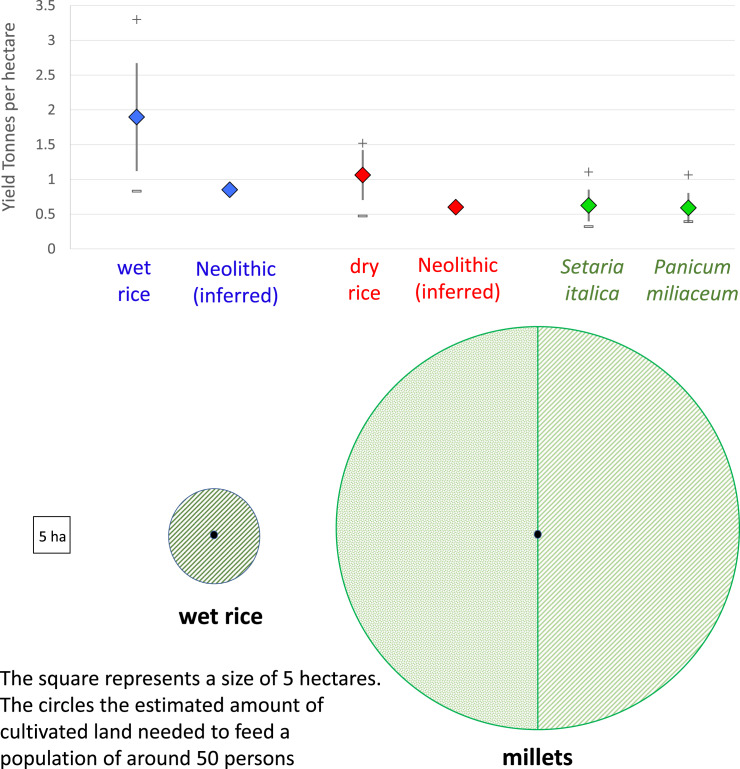
Comparative productivity of early Chinese cereals. Top: graph comparing traditional reported yields in wet rice, dry rice and millets: mean, standard deviation, maximum and minimum. Lower estimated Neolithic yields of wet and dry rice after Qin and Fuller (2019). Below: comparison of area under cultivation for a 1 ha settlement (~50 persons) for wet rice vs. millets (in the latter, half the land area is assumed to be fallow at any one time).

In terms of explaining past demic diffusion, the productivity of particular economies (e.g. cropping systems) becomes important as higher productivity and carrying capacity will lead to less frequent population fission and dispersal than less productive systems. Thus highly productive crops, such as wet rice or wheat, can support larger communities on the basis of local production, compared with much less productive systems, such as Asian or African millets (Qin & Fuller, 2019; Fuller et al., 2019). Past yields may be difficult to estimate, as this depends on land-use systems. Modern traditional yields may not be perfect analogues for earlier in prehistory because both land-use systems and crop genetic varieties have been evolving. Nevertheless, we would contend that ethnographic and historical productivity frames a general magnitude of possible past productivity. In general, wet rice is expected to produce higher yields comparatively to rainfed rice, and therefore the lower bounds of reasonable yields are provided from data on dry rice productivity. Dry rice yields, from a range of sources, average 1062 kg/ha (Fig. 1), although data from Palawan and Borneo swiddens average just 578 kg/ha, with yields as low as 229 kg/ha (Barton, 2012). The average of our compilation of wet rice yields is 1897 kg/ha. Historical data, however, indicate that about 1300 kg/ha was achieved in tenth century Japan and around 1000 kg/ha in Han Dynasty Hangzhou nearly 2000 years ago, and it was therefore concluded that 800–900 kg/ha is probably a reasonabe estimate for the Neolithic (Qin & Fuller, 2019). Based on the above estimations Neolithic rice-producing sites can be inferred to require between 6.25 and 9.75 ha of rice cultivation for every 50 persons or roughly each hectare of settled area, with a median estimate of about 8 ha of rice cultivation for each hectare of settlement area (Qin & Fuller, 2019). In contrast rainfed rice in the Neolithic is suggested to have yielded ~600 kg/ha, and unlike wet rice would require fields to be fallowed, perhaps every second or third year. This leads to the estimate that rainfed rice would have required ~35 ha of cultivated land for every 50 people, with up to half of this being under fallow at any one time (Fig. 1). This means that in absolute terms the land in the general vicinity of a settlement, e.g. within 3 km (cf. Carlstein, 1980: 172; Chisholm, 1968), would support a lower population density under a rainfed system than a wetland system. Using the aforementioned figures, and if we assume population growth rates were equivalent between dry-rice and wet-rice farming communities, then dry rice farming settlements would need to expand spatially by fission at around four times the rate of wet rice farmers.

For traditional millet production in northern China, yields can be inferred to be similar to that for dry rice (Fig. 1). The loess soils in northern China, where many early cultivation settlements were located, might have reduced the need for fallow as these soils have high potential fertility when well watered. The inherent mineral nutrients of these soils are limited primarily by their potential to absorb water. As such prehistoric millet sites are often located in the regions between the upland slopes and the softer sediments of the foothills, which provide the critical balance between water absorption and drainage (Liu et al., 2009, 2015). Ho (1975) infers, both from deductive principles and by written references to Zhou Dynasty agriculture (ca. 850 BC), that land was likely to be cleared in the first year, planted in the second and third, and then left fallow again for a year (Ho, 1975: 49–54). Late Shang Dynasty (1250–1046 BC) oracle bone inscriptions also suggest regular fallowing, with fallowed land used for hunting in years without cultivation (Peng, 1989). Under such a rotation we estimate that between 30 and 36 hectares of cultivated land was needed for 50 people on the most productive loess land (Qin & Fuller, 2019). For self-sufficient early millet farming communities, a 3 km catchment area around a settlement could then support around 4000 people at a maximum. However, typical Neolithic millet carrying capacity might be actually less, perhaps half the above estimate, because not all land is equally fertile. Less well-watered lands might need to be rested every other year, increasing land needs and lowering carrying capacity. As millet cultivation was taken beyond the loess plateau, and especially onto lower fertility soils in the sub-tropics and tropics, or less well-watered areas to the northwest, fallows are likely to have increased to two years out of three or even more. Thus as millet cultivation spread beyond its core area in the loess plateau it would probably require increasing land areas to maintain the same levels of productivity to support similarly sized communities. The lower land requirements for rice equate to much higher potential population densities, with less frequent population fission (splitting) and expansion for wet rice farmers compared with either dry rice or millet farmers. In other words, millet agriculture tends to *push* populations towards outward expansion in contrast to early wetland rice farming (Qin & Fuller, 2019). A similar point has been made with regards to Southeast Asia, that it was less productive rainfed rice farming that drove the initial Neolithic expansion (Fuller, 2020).

**Figure 2.**
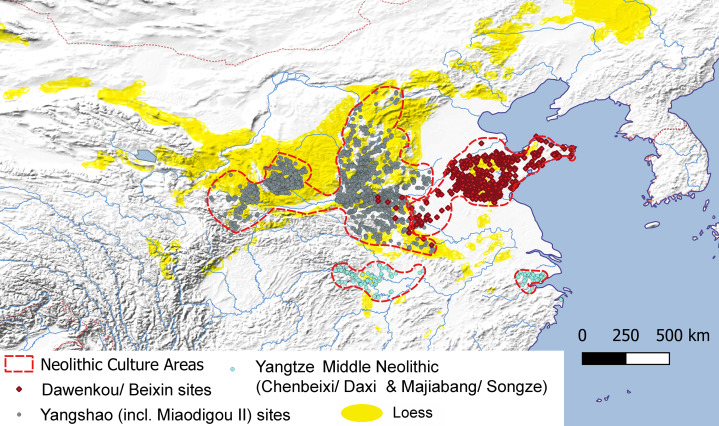
The distribution of major Neolithic culture complexes (c. 5000–3000 BC) in central and northern China. Site distribution data after Hosner et al. (2016).

What the above line of reasoning leads us to conclude is that we might expect millet farmers to expand at a greater rate than the wetland farmers of the Neolithic Yangtze. This expectation is reflected in the much larger geographical areas attributed to established millet cultures than to early rice farming cultures (Fig. 2), such as the Yangshao/Maiodogou II (~430,000 km^2^) and Beixin/Dawenkou (~120,000 km^2^) area vs. those of the Middle Neolithic Yangtze, such as Majiabang-Songze (~14,000 km^2^) or Chenbeixi/ Daxi (~65,000 km^2^).

## The pull of domestication: the first productivity transitions

The above yield estimates are based on fully domesticated millets, and it is therefore important to consider how productivity changed during the domestication process. Domestication is a set of changes in the morphology (and underlying genetics) of plants that make them better suited to human cultivation and harvesting, and which also improve productivity. As it has become increasingly apparent that documented cereal domestication processes were protracted, taking millennia (e.g. Fuller et al., 2014; Allaby et al., 2017), we need to consider how harvesting returns for early crops also improved over this period. This improvement is itself an inherent pull-factor for domestication (Fuller, 2020), and a process that would have facilitated increasing population density. Domestication resulted in increased productivity (yield) per plant and per area harvested, while at the same time requiring increased labour inputs (Fuller et al., 2010, 2016). In addition, cultural changes that took advantage of these changes, such as harvesting technologies, may well have increased relative productivity further.

We therefore can consider how key domestication traits resulted in higher productivity. Experimental work and functional genetic studies are well documented for a few species such as rice. Based on experimental data it is possible to estimate how key morphological changes with domestication increased the average productivity of rice over its wild progenitor by around ~366% (Fuller, 2020). The basis for these estimates is summarised in Table 1, together with comparable estimates where possible for *Setaria* and *Panicum.* Where observational data for estimates are not available for the millets, we have taken estimates from rice. Altogether this leads to an estimate of a 1180% improvement in yields through domestication for *S. italica* and a 546% improvement for *P. miliaceum.* Of course genetic changes were gradual, as alleles associated with domestication slowly accumulated and moved towards fixation. Thus improvements in the productivity of these cultivated millets were slow, spread across the entirety of the domestication process and continued after. Indeed, measurable changes in grain dimensions suggest that roughly half the total amount of change in grain width took place after 4000 BC and therefore probably after domestication, as inferred by other traits (see below). The wild forms of *Panicum* were probably better yielding in terms of overall grain weight than *Setaria*. However, greater increases in yield in *Setaria,* through the increase number of grains per panicle, combined with a relative greater percentage increase in grain size, meant that at some point in the past *Setaria* overtook *Panicum* in terms of yields. As discussed below, this probably occurred around 4300–3800 BC (cf. He et al., 2022a). This observation may help to explain why it is that most earlier millet cultivation (in the pre-Yangshao period) had a greater emphasis on *Panicum* in contrast to the dominance of *Setaria* in later periods (Qin, 2012; Stevens & Fuller, 2017), rather than relating to other explanations, such as climatic deterioration (He et al., 2022a; Yang et al., 2022a) or population pressure (Li, Y. et al., 2021). Taking these estimates of changing yields per hectare, it can be seen how the domestication and post-domestication processes themselves would have facilitated higher population densities, relating to both increasingly widespread sedentism and increased maximum site size.

**Table 1.** Yield increases resulting from the evolution of domestication traits, estimated for rice and millets. New estimates for this paper are indicated in italics

Trait	*Oryza rufipogon* to *O. sativa* (percentage increase)	*Setaria viridis* to *S. italica* (percentage increase)	*Panicum ruderale* to *P. miliaceum* (percentage increase)
Seed weight (g)	0.0188 → 0.0232 (23.4%)^1^	0.0013 → 0.003 (130.8%)^7^	0.0039 → 0.0059 (51.3%)^14^
Grains per panicle	47.5 → 110.6 (132.8%)^1^	500^8^ → 5000^9^ (900%)	*380* → 1500^15^ (294.7%)
Closed panicle (or denser panicle)	20% → 30% (50%)^2^	n/a (?)	(*estimated 50%)*^16^
Immature harvest to mature harvest	60% → 90% (50%)^3^	40%^10^→ 60%^11^ (50%)	(*estimated 50%)*
Compact erect growth habit	20 g → 42 g (of grains per plant) (110%)^4^	(*estimated 100%)*	(*estimated 100%)*
Yield rate estimate kcal/hour	90^5^ → 330 (366.2%)^6^	21.06^12^→ *248.67* (1180%)	*30* → *163.8* (546%)
Yield estimate kg/ha	232 → 850 (366%)^6^	*50.814* → 600^13^ (1180%)	*109.9* → 600^13^ (546%)

Sources: ^1^Morishima et al. (1961); ^2^Ishii et al. (2013); ^3^Fuller et al. (2007); ^4^Tan et al. (2008); ^5^Lu (2006); ^6^Fuller (2020); ^7^Lu (2002); ^8^upper limit reported by Douglas et al. (1985), lower estimates in Lu (2002); ^9^median of range reported by Lu (2002); ^10^Lu (2002); ^11^Song et al. (2013); ^12^yields from Lu (1998) multiplied by 351 kcal/g from USDA databases; ^13^rounded estimate from Figure 1; ^14^Eberlein et al. (1990); ^15^Rajput et al. (2016); ^16^compact/closed panicle described as domestication trait in Li et al. (2021). Other values *in italics*, estimated here, require observational studies.

**Figure 3.**
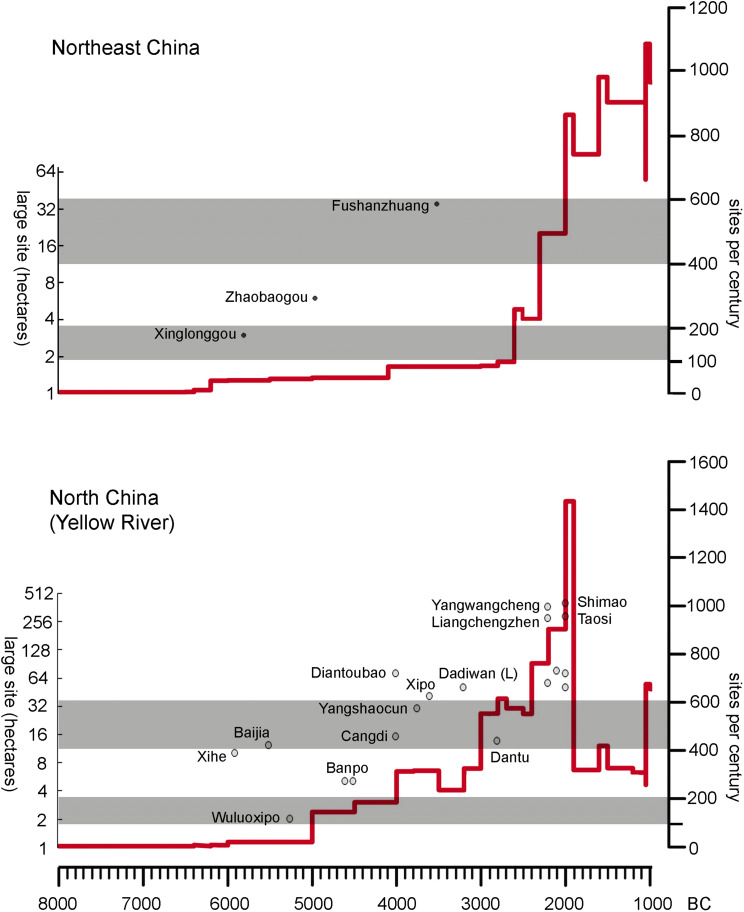
Proxies for population growth and population density growth for northern and northeastern China. Solid lines represent an estimate of number of sites occupied per century (after Leipe et al., 2019), shown together with selected representative large sites (large for their period and region, plotted against estimated median age). (For estimated site sizes see Table S1.)

While it is not straightforward to estimate past populations or population densities, some observations of site numbers over time and the general range site sizes can be informative (Fig. 3). Previous compilations of site counts over time indicate an approximately geometric growth of site numbers per century over the course of the Neolithic (Wagner et al., 2013; Hosner et al., 2016; Stevens & Fuller, 2017; Leipe et al., 2019). The number of sites not only increased over the course of the Neolithic, but increased at a ‘quasi-exponential’ rate, with notable upturns in the number of sites between around 5000 and 4000 BC, most notably in the later part of the Early Yangshao period; further increases are seen from 3000 BC particularly around 2500–2000 BC (Leipe et al., 2019). For part of the Central Plains Ren et al. (2021) have inferred periods of quicker or slower population growth from the summed probability of radiocarbon dates. They infer a greater than logarithmic population increase from 6000 to 5600 BC, followed by a period from ~5400 to 4400 BC when population appears to have declined, with a subsequent major period of rapid population growth seen from 3500 to 3000 BC, a period of decline during ~2800–2300 and period of population increase from ~2000 BC. A similar pattern after 3500 BC is probably also evidence for the Ganqing region to the west (He et al., 2022b). In northeast China increases in site counts are more subtle until a notable population rise is noted from 2500 to 2000 BC (Fig. 3; Leipe et al., 2019). Over the same period the size of the largest sites also increased (Fig. 3). Prior to 5000 BC larger sites are generally 2–3 ha, with the largest sites of 10–12 ha recorded over a wide area from the Laoguantai Culture (Baijia site) of the Wei Valley to the Houli Culture of Shandong in the east (Xihe site). Towards the transition to the Middle Yangshao, ca. 4000 BC, the largest sites range in size from ~15 to 70 ha, while in the Longshan horizon, 2500–2000 BC, large sites covering hundreds of hectares first appear, which can be regarded as early urban centres.

It is worth reflecting on what these site sizes mean in relation to millet productivities. Based on the estimated productivity of millet falling between 500 and 650 kg/ha with one-half to one-third of land fallowed at any one time, and assuming self-sufficiency, the maximum population that could be sustained on cultivation within a 3 km radius (a working day's walk: see Chisholm 1968; Carlstein, 1980) of settlement would be around 2000 people, equivalent to perhaps a 40 ha site (Qin & Fuller, 2019). Of course, such an estimate would mean that almost no land (of the ~2950 ha surrounding a site) was left for woodland, forcing hunting and other activities, such as collecting fire wood, to locations much further away. Nevertheless, it suggests that in general most sites through the Yangshao period would have been potentially self-sufficient. As populations reached the carrying capacity of the surrounding landscape this resulted in community fission, followed by outward expansion and the creation of new farming settlements where none had previously existed, potentially at some distance beyond the original settlement.

Population sizes supported by millet farming could potentially have been higher if we assume higher millet yields or a reduction in the amount of fallow needed. Some researchers have speculated that millet crops may have been manured, which would conceivably have improved yields and removed regular fallowing. Wang et al. (2018) postulated on the basis of elevated *δ*^15^N isotopes levels recorded for archaeological millets from sites (3500–1500 BC) in Shaanxi, north China, that crops were manured at least during the Late Neolithic. However, this study relied on figures developed for wheat and barley in Europe and contrasts with background vegetation inferred from archaeological herbivores. The study of Dadiwan isotopes is more reliable as it included sampling across the food chain (millets, pigs, humans) from the same site, as well as a small baseline study of modern vegetation and millets grown in the region (Yang et al., 2022b). This study suggested that during Yangshao period pigs (and perhaps people) fed on millet (or millet food waste) provided manure back to the millet fields, raising the *δ*^15^N above that of background vegetation. Such intensification may well have been more sustainable and would have reduced fallow requirements, increasing carrying capacity. Nevertheless more research is needed to determine how widespread such practices were across space and time in Neolithic north China. Further baseline research in understanding the determinants of *δ*^15^N in millets is also needed. The photosynthesis of C_4_ millets, however, can be expected to affect nitrogen isotope levels through reduced stomatal opening and increased water use efficiency. While some studies on C_4_ millets, including *S. italica* and African *Eleusine* and *Pennisetum* found an initial tentative relationship between *δ*^15^N levels and the amount of watering or rainfall, a conclusive correlation between *δ*^15^N levels and water availability was not demonstrated, and further work is still required (Lightfoot et al., 2020). Similarly, millet accessions collected from Africa indicate a correlation of higher *δ*^15^N values with higher rainfall (Reid et al., 2018), although another experiment on *Pennisetum glaucum* failed to find a strong impact of watering on nitrogen isotopes (Sanborn et al., 2021). Thus, presently while a combination of manuring, water availability, soil type and soil chemistry probably affects *δ*^15^N values, further work is necessary to clarify this situation and how such values might pertain to past cropping regimes. As discussed by Ho (1975: 50–51) inherent fertility in loess soils is limited by water availability, and wetter conditions with reduced need of fallowing and yields of ~650 kg/ha, could potentially have supported populations of ~7000 people, assuming cultivation within a 3 km radius of the site. With higher yields (~800 kg/ha), but alternative years of fallow, an estimated carrying capacity for the same area of land might be ~5000. This suggests that as large sites (>40 ha) appeared, networks of smaller sites must have been supplying surplus production to help support the larger centres. Ultimately urbanism is supported by such supply chains, as are the growing number of non-agricultural specialists that come with urbanisation. In this sense urbanisation processes were probably underway in some areas in the later Yangshao period commencing in the fourth millennium BC, with emergent regional settlement hierarchies and regional centres appearing by the Late Yangshao (3500–3000 BC; Liu & Chen, 2012). However, the initiation and continuation of urbanisation into later periods, in terms of driving forces is still poorly understood (Jing et al., 2013).

## Millet origins: evidence for domestication trajectories

**Figure 4.**
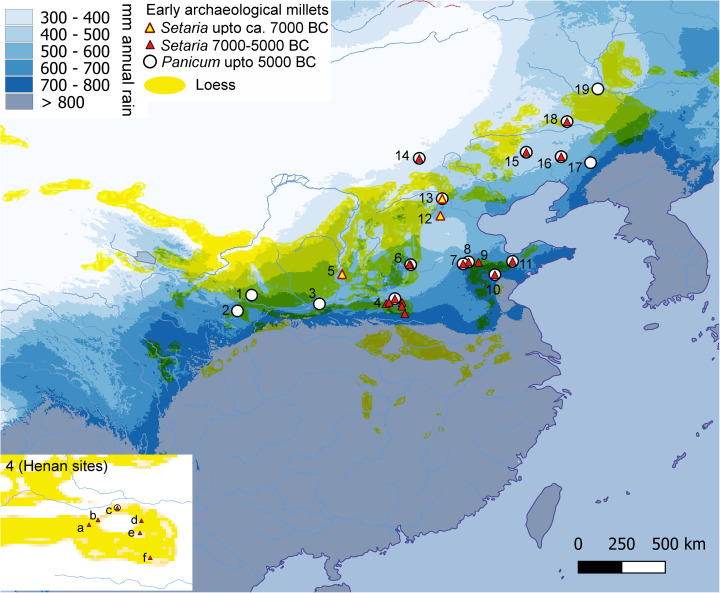
Distribution early archaeological millets across northern China, in relation to rainfall zones and the Chinese loess distribution (wind-blown loess only). Sites numbered: 1, Dadiwan; 2, Lixian VII; 3, Bajia; 4a, Fudian; 4b, Wuluoxipo; 4c, Zhuzhai; 4d, Shawoli; 4e, Peiligang; 4f, Dingzhuang; 5, Shizitan IX; 6, Cishan and Niuwabao; 7, Yuezhuang; 8, Zhangmatun; 9, Xihe; 10, Bainbiandong; 11, Qianbuxia; 12, Nanzhuangtou; 13, Donghulin; 14, Xinglong; 15, Xinglonggou; 16, Fuxin Jiajiagou and Tachiyingzi; 17, Xinle; 18, Mangha; 19, Houtaomuga. Rainfall data are the mean annual rainfall from 1970 to 2000 derived from WorldClim 2.1 (Fick and Hijmans 2017; worldclim.org). (For site information, see Table S2.)

**Figure 5.**
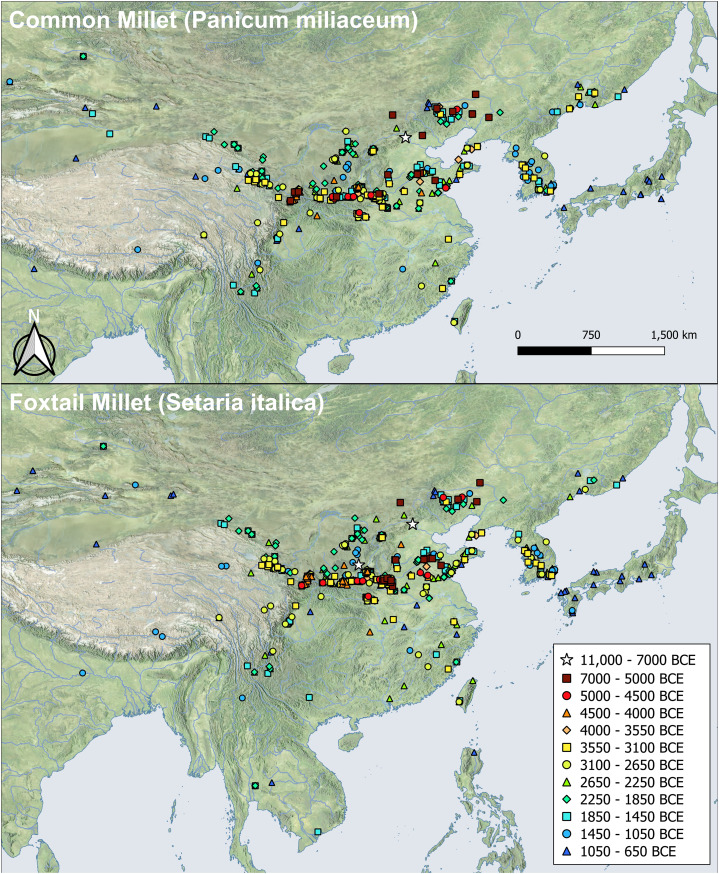
Map of the distribution of millet crop remains in East Asia up to 650 BCE for China and adjacent regions. (For site information, see Table S3.)

A key issue in the development towards sedentism and increased population density is where and when millets came to be cultivated and underwent domestication. It seems clear that well-developed Middle Neolithic cultures, such as the Yangshao, Dawenkou or Hongshan, had millet-centric agricultural systems, and these were quite widely established by 4500–3500 BC across the Yellow River basin and the Loess Plateau region in northern China (e.g. Liu & Chen, 2012; Stevens & Fuller, 2017). Therefore, attention has tended to turn to a series of ‘pre-Yangshao’ cultures distributed in this area, mainly in the Chinese Loess Plateau zone that date variously between 6500 and 5000 BC, and this has produced some evidence for the presence of millets (Fig. 4). Thus finds are distributed in the ecotonal region where rainfall for the summer monsoon decreases (mean rainfall from 400 to 700 mm in modern times), constituting a transition from dominant temperate woodland to grassland steppe. The wild relatives of the domesticated millets are known to be *Setaria viridis* (Jia et al., 2013; He et al., 2023) and ancestral relatives of the *P. miliaceum* subsp. *ruderale* group (Xu et al., 2019b; Li, C. et al., 2021). Both groups include feral and weedy forms, and gene flow from domesticated forms makes these imperfect analogues for the original progenitors (Stevens et al., 2021). Both *S. viridis* and *P. miliaceum* ssp. *ruderale* occur widely as anthropogenic weeds and less often in true wild habitats, which means that their current geography provides limited evidence for where domestication took place. Nevertheless, the earliest evidence for exploitation of presumably wild millets occurs across these similar semi-arid zones in the loess regions. There is evidence for the use of *Setaria* and *Echinochloa* by terminal Pleistocene foragers at Shizitan, southwest Shanxi (Bestel et al., 2014). Starch grain evidence from stone tools suggests millet consumption in the eastern loess plateau sites in the early Holocene, including Nanzhuangtou, Zhuannian and Donghulin (9500–7500 BC; Fig. 4; Yang et al., 2012, 2015). Recently, millet grains from Donghulin have been reported to include both clear wild-type foxtail millet and grains with a ‘domesticated shape’ (Zhao et al., 2020). There is still a limited number of sites that have produced millet archaeobotanical remains older than 5000 BC, but over the subsequent millennia finds of both foxtail millet and broomcorn millet are widespread and increasingly so across northern and northeast China from 4000 to 3500 BC, to the Russian Far East (Li et al., 2020; Sergusheva & Vostretsov, 2009; Sergusheva et al., 2022) and the Korean peninsula from 3500 to 3000 BC (Crawford & Lee, 2003; Lee, 2011; Stevens & Fuller, 2017; Stevens et al., 2022; Fig. 5).

**Figure 6.**
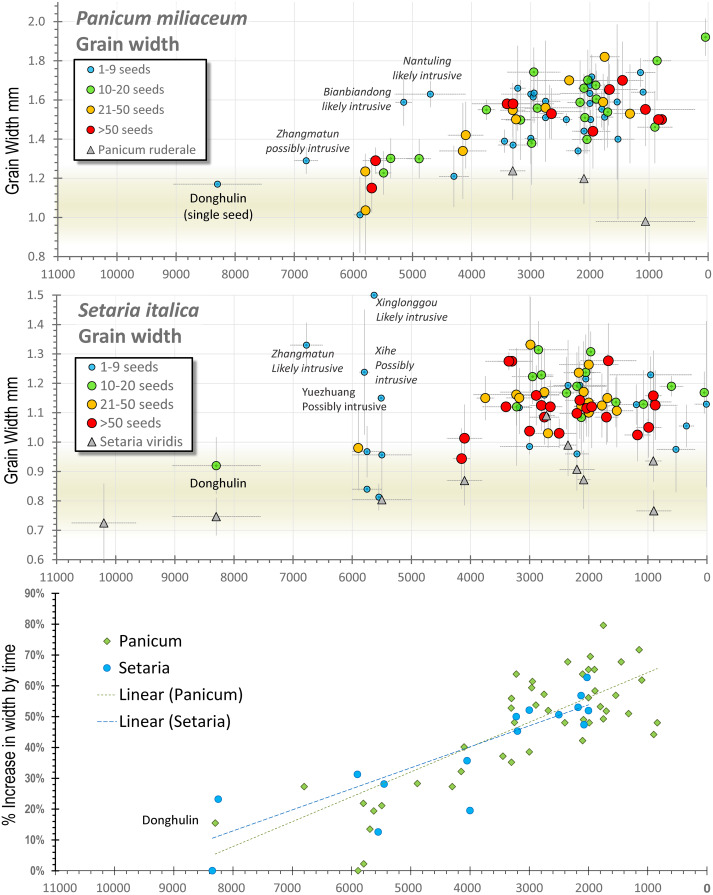
Grain size data and millet domestication, plotting assemblage means and standard deviations for grain width against median age estimates. The lower graph compares the mean increase in size as a percentage increase, comparing trends in *Panicum* and *Setaria*. For Donghulin (DHL) two shape categories of *Setaria* are separated, with the inferred *Setaria viridis* wild-type plotted separately (after Zhao et al., 2020). *Panicum miliaceum* data (*n =* 1210) from Stevens et al. (2021) with the addition of DHL (Zhao et al., 2020). *Setaria* data (*n =* 791) include data from Lee et al. (2007), Qin and Fuller (2009), Deng et al. (2015), Bestel et al. (2018) and Zhao et al. (2020) and additional data from the authors. (For data, see Table S4.)

While limited, the evidence for change in grain shape and size in the Chinese millets indicates an evolutionary process, providing a potential timeline for their initial domestication. In the study of other cereal domestications, such as those of wheat, barley, rice or pearl millet (e.g. Fuller et al., 2014, 2018, 2021), it is apparent that changes in grain size and shape began around the same time as changes in other domestication traits, such as loss of natural seed dispersal. However, whereas no further change can occur once the transition from shattering to non-shattering reaches 100%, grain size often continues to change after non-shattering has been fixed in many cereals, resulting from both continued selection under cultivation for grain size and also the spread of crops into new environments (Fuller et al., 2018, 2021). It is probable therefore that some regional and even local variation should be expected in grain size. In particular during the post-domestication period, following the emergence of non-shattering and greater density panicles, such selection for grain size might be further modified by deliberate human selection. Such explanations are in accordance with the continued increased average grain size seen in both foxtail and broomcorn millet between 4000 and 2000 BC (Fig. 6, lower graph). So far for the Chinese millets, morphometric data are too sparse and geographically dispersed to provide separate regional assessments of such changes. Furthermore, placing time limits on the domestication period is problematic. For *P. miliaceum* grain size change data indicate an initial period of size and shape change starting around 6000 BC, with a clear increase in size seen after 4000 BC (Fig. 6; cf. Stevens et al., 2021). For *Setaria* clear size and shape change indicative of cultivation is seen around 9050–7550 BC at Donghulin (Zhao et al., 2020). However, whether this represents the start of continuous cultivation leading to domestication is unknown, especially given the small number of sites with millets between 5200 and 4200 BC, only after which a clear size increase is seen (Fig. 6). Thus on size change we can at best posit an era of pre-domestication cultivation for *Panicum* that includes the sixth millennium BC which presumably, as seen for other cereal crops, starts earlier (cf. Allaby et al., 2017). While evidence for the transition from shattering to non-shattering plants in millets is absent, the use of stone and ceramic harvesting knives begins around 4600–4300 BC with a notable increase in stone harvesting knives after 4000 BC (Luo, 2007a). While the relationship of such knives to non-shattering panicles is unknown, their appearance and subsequent increasing frequency would be conducive to the dominance of non-shattering forms, as well as potentially an enlarged panicle. Finally, as noted above, the change in Central China from *P. miliaceum* to *S. italica* occurs around 4300–3700 BC (He et al., 2022a; Li, Y. et al., 2021), and slightly later, c. 3500 BC, on the Western Loess Plateau (Yang et al., 2022a). This change can potentially be attributed to a point in time in which the evolution of a denser and larger panicle in *S. italica*, compared with the small panicle of *S. viridis*, provided higher yields than *P. miliaceum*. Taken together the evidence suggests that pre-domestication cultivation began at least around 8300 BC, with non-shattering forms dominating before the end of the Early Yangshao (5000–4000 BC), with panicle enlargement in *Setaria* probably increasing its productivity over *Panicum* by around 4000 BC. In terms of increased grain size, a size increase of about quarter to half of the mean size (+30% width) took place by 5000 BC, while the other half (up to +60% width) took place thereafter (Fig. 6 lower graph). For this trait at least (and probably others e.g. panicle enlargement) yield improvements approximately doubled over the course of the Yangshao era. In light of the issues raised above this implies continued productivity improvement throughout this period, alongside those increases brought about through cultural practices (tillage, irrigation, manuring and harvesting methods) and through expansion of cultivated area.

The growing importance of millets in Neolithic northern China is reflected in human dietary evidence inferred from carbon isotopes (^13^C). A large body of isotope data has become available in recent years (Liu et al., 2021) showing that while most populations after 4000 BC are consuming large amounts of millet, earlier sites show a greater diversity. In the Early Yangshao period, sites such as Gouwan (mean *δ*^13^C −14.4‰) and Banpo (mean *δ*^13^C −14.8‰), both in the broad Central Plains region, suggest a mixed C_3_ and C_4_ plant-based diet, which we can infer represents a considerable dependence on wild plant sources, and especially in the case of Gouwan, rice, alongside millet. Nevertheless, sites that we would place in the pre-domestication cultivation period show considerable variation in human isotopic values, with Xinglongwa and Xinglonggou, Inner Mongolia in the northeast being strongly C_4_ (mean *δ*^13^C of >12‰; Liu & Reid, 2020), whereas Xiaojingshan, a Houli culture site in Shandong, is more tilted towards C_3_ (mean *δ*^13^C of −17.8‰; Hu et al., 2009). It is worth noting here though that the site of Xinglong, 5700–5600 BC, in northern Hebei, on the Inner Mongolian border, has considerable numbers of gathered edible species, including those of C_4_ and intermediate C_3_–C_4_ species (Amaranthaceae/Chenopodiaceae) which often outnumber millets (cf. Qiu et al., 2023). Nevertheless, despite these variations the trend over China is clearly one in which people consumed a greater proportion of millets over time.

It is not necessarily the case that all regions participated in this process equally or that both millet species were evolving together in the same region. It is probably better envisioned therefore as a ‘geographical mosaic of coevolution’ (*sensu* Thompson, 2005) in which some localities saw clear selection for domestication while others did not, while the protracted process allowed for gene flow, notably through intercultural exchanges (see Allaby et al., 2022). It is increasingly evident for western Asia that a very wide region or multiple sub-regions participated in the processes of domestication (e.g. Fuller et al., 2011; Allaby et al., 2017), and a similar macro-regional perspective can be suggested for northern China. This highlights the need for more empirical evidence from further geographically widely distributed sites, as well as the need to consider how millet cultivation was entangled with local landscapes, settlement contexts and increasing integration of other taxa, such as domesticated animals, the dog and the pig.

## From dog domestication to neolithicisation

Dogs are a domesticate of the Palaeolithic, but are nevertheless typical components of Neolithic sedentism. Genomic data have been argued to support dual origins, with separate domestication processes in eastern and western Eurasian in the later Pleistocene (Frantz et al., 2016), but recently genetics have been argued to also fit with a single early domestication process and subsequent differentiation across Eurasia (Bergström et al., 2020). Bergström et al. (2020) estimate that domestication took place around 20,000 years ago. Archaeological finds of apparent dogs at a number of sites from Western Europe to Russia suggest that domestication of wolves had already taken place by the time dog-like specimens are found, with early finds from ~9 sites dated between 16,000 and 13,000 BP (Perri, 2016; Morey and Jaeger, 2017). In terms of process it is inferred that some wolf lineages evolved to be more human tolerant and commensal, later co-habiting with and hunting alongside people (Larson & Fuller, 2014; Morey & Jaeger, 2017). Nevertheless, what is clear is that dogs, associated with hunter–gatherers, were established across temperate Eurasia well before agriculture. So far in China dogs have been reported only from a few Early Holocene, probably pre-agricultural sites, including Nanzhuangtou (ca. 8100 BC), Bianbiandong (ca. 7400 BC) and Zhangmatun (ca. 7000 BC) (Liu & Chen, 2012: 98; Song et al., 2019). Despite this, dogs become a fixed feature of settlements from the Neolithic period onwards, and are widely reported from sites associated with early cultivation (7000–5500 BC), e.g. Jiahu, Cishan, Dadiwan and Xinglonggou (Liu & Chen, 2012; Liu et al., 2012; Song et al., 2019). If Palaeolithic dogs are few and far between, Neolithic dogs are a typical aspect of early Neolithic villages, regardless of whether one considers them of western or eastern Eurasian origin. To some extent this may be attributed to the effects of sedentism, promoting increased dog population density, like those that affected human population growth. Readily available food and reduced mobility made it increasingly likely that puppies would survive to reproductive maturity. Furthermore sedentary sites created improved depositional conditions for dog bone preservation.

The increased consumption by dogs of food from farming diets can be expected to include more starchy foods, and this is supported by evidence that dogs, or at least most lineages of domesticated dogs, have increased amylase gene (*AMY2B*) copy number (Arendt et al., 2016; Pajic et al., 2019), and this is evident in ancient dog genomes in which higher amylase gene copy numbers occur in later farming-associated dog remains (Bergström et al., 2020). In humans, increased amylase gene copy number is thought to be associated with increasingly starch-rich diets, and provides a genetic contrast between anatomically modern humans and Neanderthals or great apes (Inchley et al., 2016). It is estimated that modern humans started to evolve more amylase (higher *AMY2B* copy number) from about 450,000 years ago (Inchley et al., 2016). Similarly, in comparison with wolves which have typically two *AMY2B* genes, most dog breeds have more (from three to eight; Arendt et al., 2016; Bergström et al., 2020). Exceptions to this are a few dog breeds with the ancestral two *AMY2B* copies, such as Australian dingos and Greenland husky-like dogs, associated with human hunter–gatherer contexts, as well as some African village dogs (Arendt et al., 2016; Bergström et al., 2020).

Taken together what these genetic patterns suggest is that whereas dogs were undoubtedly widely distributed in the Palaeolithic and subsequent Mesolithic, most dogs around today are descended from those populations that expanded during the Neolithic that were adapting to starch-rich, cereal-based lifestyles. In Europe and western Eurasia, ancient dog genomes indicate a major turnover occurred with the spread of farming as Mesolithic dog haplotypes were mostly replaced by new farming-associated dog lineages from the Near East (Ollivier et al., 2018; Bergström et al., 2020). Ancient dog genomes from Neolithic China indicate the predominance of a haplogroup that began to diversify 9500–8500 years ago with a major increase in effective population around 7500 years ago (Zhang et al., 2020). This fits with a major turnover in dogs in Neolithic China starting 9500–7500 years ago, the era of early millet cultivation. Although this haplogroup was largely replaced across China over the past 2000 years, it is ancestral to the predominant dog lineages of Southeast Asia and the Pacific, as expected from archaeological inferences of dog dispersal with Neolithic migrations out of China.

Genetic evidence from modern East Asian dog breeds supports the expansion and differentiation of most dog types since the Neolithic. Yang et al. (2017) used modern genomic data to explore population structure and differentiation among East Asia dogs, including the Chinese chow-chow and shar-pei breeds, the Japanese Akita, Siberian huskies and Tibetan mastiffs amongst others. These breeds formed a distinct sub-population between European dog breeds and wolves. Among the East Asian clade group, chow-chow dogs appear to have differentiated first, and, based on genetic coalescence within this group, the divergence of chow-chows from other East Asian breeds was estimated to have taken place around 8300 years ago (Yang et al., 2017). While genetic estimates of age are notoriously imprecise, this nevertheless fits with the period of the Pre-Yangshao cultures that are associated with pre-domestication cultivation of millets and emergent sedentism. The East Asian dog genomes also imply that Neolithic or post-Neolithic dispersal of dogs, perhaps outwards from the Yellow River region, included the dogs introduced into Tibet, into Siberian human societies and into Jomon Japan, where the Akita and Shiba breeds have been compared with dog skeletons from Jomon period dog burials (Tonoike et al., 2015).

**Figure 7.**
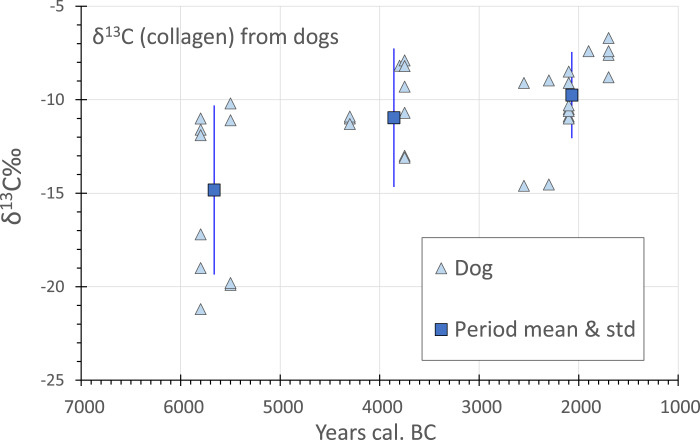
Carbon 13 measurements from archaeological dog remains from Chinese sites, plotted against median age estimates (*n =* 37). Means and standard deviations are plotted for three period clusters. Sites and sources: Xinglonggou 1 (Liu et al., 2012), Dadiwan (Barton et al., 2009), Wayaogou (Chen et al., 2016a), Xipo (Pechenkina et al., 2005), Kangjia (Pechenkina et al., 2005), Wadian (Chen et al., 2016b), Xinglonggou III (Liu et al., 2012) and Zhangdeng (Hou et al., 2013). (Raw data in Table S5.)

Returning to the zooarchaeological record in China, stable isotope studies highlight that over the course of the Neolithic dogs ate an increasingly cultural diet that included millets (Fig. 7). Already in the Early Neolithic, at Dadiwan and Xinglonggou, about half the sampled dogs consumed C_4_-enriched diets (ca. −10‰), suggesting that they were fed on millets, potentially through human food leftovers, while the remaining dogs had a more C_3_-focused diet (ca. −20‰), consistent with eating wild game, etc. Records from around a millennium later, where data are available, show no dogs to have produced *δ*^13^C below −15‰, indicating that all had a substantial intake of millet foods. It is just such a pattern that can be expected to correlate with the evolution of increased starch consumption and represents the increased integration of dogs with millet farming (e.g. Barton et al., 2009).

## Pig domestication and sedentarisation

Unlike the bovines and caprines domesticated in Western Asia that lent themselves to mobile pastoralism, the early farmyard animal of the Chinese Neolithic was the pig, which are more readily adapted to fully sedentary conditions. While wild boars are widespread throughout China, genetic data suggest that the majority of these wild boar populations did not contribute to modern and ancient Chinese domesticated lineages (Larson et al., 2010). In contrast to zooarchaeological evidence that implies potentially multiple pig domestications in China, genetic evidence suggests potentially far fewer domestication events (Lander et al., 2020). Ancient DNA data from Chinese pigs support domestication in the middle Yellow River region from which the majority of subsequent pig diversity descends (Larson et al., 2010; Xiang et al., 2017). Distinct genotypes do appear in northeast China, some of which are now extirpated, with evidence for gene flow between the Yellow River and northeast China, with Yellow River pig lineages coming to dominate. This was originally hypothesised as possibly pointing to a separate domestication of pigs in northeast China, but might equally relate to introgressions of local boars into incoming domesticated pigs (Xiang et al., 2017). A recent paper has argued that new data are more in keeping with the second scenario rather than northeast China representing an independent domestication centre (Wang et al., 2022).

**Figure 8.**
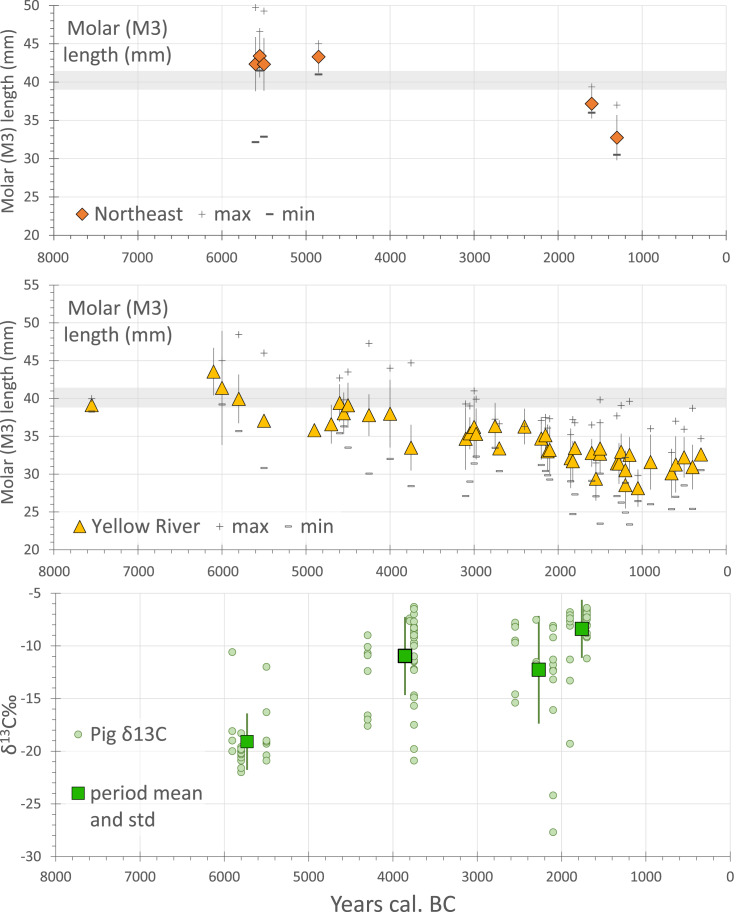
Changes representative of pig domestication in northern China. The top two graphs chart change of M3 length of Holocene pigs in Northeast China (*n =* 111) and the Yellow River valley (*n =* 1183). The *y-*axis represents the average length (mm) of M3 whereas the *x-*axis is the estimated median date (BC) for each assemblage. Raw data from Luo (2007b), Wang et al. (2015) and Song et al. (2019). Lower graph charts measure carbon-13 isotopes from pig bones (collagen) against median age estimate (*n =* 115); period means calculated for four groups. Sites and sources: Yuezhuang (Hu et al., 2008), Xinglonggou 1 (Liu et al., 2012), Dadiwan (Barton et al., 2009), Wayaogou (Chen et al., 2016a), Xipo (Pechenkina et al., 2005), Xinglonggou II (Liu et al., 2012), Dongying (Chen et al., 2016a), Kangjia (Pechenkina et al., 2005), Wadian (Chen et al., 2016b), Xinglonggou III (Liu et al., 2012) and Zhangdeng (Hou et al., 2013). (For size data, see Table S6; for isotopic data see Table S5).

While there remain some claims for a southern Yangtze pig domestication process as well, in the context of millet-growing China we can infer pig domestication from genetics only once in the middle Yellow River region. Zooarchaeological evidence can be used to examine this pathway to domestication in two ways, either from evidence for human management or from morphological change. Evidence for human management comes in the form of age:sex distributions that show culling patterns with higher levels of immature individuals, as reported from some pre-Yangshao cultures including Xinglongwa, Cishan and Dadiwan (e.g. Barton et al., 2009; Hongo et al., 2021; Yuan et al., 2008). Prolonged management, however, can be expected to result in morphological indicators of domestication, such as size decrease. Available data from the Yellow River region provides a clear case of size decrease, as seen mainly through dental morphology (Fig. 8; see also Cucchi et al., 2016), with a marked decline in size from 6500 to 5500 BC, followed by a longer continued trend in size decrease. However, data from northeastern China are too limited at present to assess any potential change in wild pig populations prior to the arrival of pigs from the Middle Yellow River Basin and Central Plains.

Another indicator of the close relationship of human and pigs can be taken from pig dietary evidence, i.e. isotopic evidence for a millet (C_4_) enriched diet (Fig. 8, bottom graph). What is notable of the stable isotope data from pigs is that most pigs from populations older than 5400 BC still have a diet with significant consumption of C_3_ plants, consistent with the consumption of wild foods (i.e. *δ*^13^C around −20‰), with just a small number of specimens being enriched in millet consumption (*δ*^13^C around −10‰). As vegetation dominated by C_4_ plants is relatively rare in north China, and wild boars are expected to consume roots, fallen fruits and acorns, etc., with a more C_3_ diet, the heightened C_4_ signature in archaeological specimens suggests that pigs were eating millets either from human food waste or through feeding by people growing millets. In the Yangshao period, after ca. 4500 BC, most isotopic data from pigs suggest heavy consumption of millets, with gradually fewer pigs having access to a C_3_ rich diet over time (Fig. 6). Taken together the data suggest that when pigs were initially managed and undergoing size change (ca. 6000 BC) they were still subsisting by a larger degree of free-range foraging, but after 4500–4300 BC they became increasingly reliant on human settlements for their food sources, resulting in a millet-enriched diet, with a lower dependence on surrounding resources (Liu & Jones, 2014). This probably also reflects the expansion of millet cultivation that would have reduced areas of wild land for growing pig herds to forage. Yangshao pigs, from at least 4300 BC, can be regarded as strongly sedentary, although the shift to a more millet-focused diet in them lagged somewhat behind that in dogs and humans. It remains unclear, however, whether the gap in data from 5400 to 4300 BC potentially indicates discontinuity in the pig populations in some regions.

We can also ask whether the shift to more millet consumption for pigs can be expected to result in higher starch consumption and selection for higher amylase gene copy number, as has been noted for dogs. In the case of pigs, it is plausible that wild boar, as omnivores with nuts and roots in their diet, may have already been pre-adapted to higher starch consumption. Nevertheless, in their survey across mammals, Pajic et al. (2019), note that pigs, alongside dogs, humans and commensal rodents (rats and mice) have higher *AMY2B* copy number consistent with an increase in situations of domestication. However, one study directly comparing wild boar and pigs found relatively high copy number in both, and no significant difference (Yoshidomi et al., 2021). The comparisons between Japanese wild boar and modern pig breeds, however, may not reflect the differences between ancestral wild boars of China and their pig descendants, which calls for further assessment in archaeological pig genomes. At present, nevertheless, we can infer that wild boar were pre-adapted to the starch-rich diets of their domestication association with humans, as they entered the settings of millet pre-domestication cultivation, resulting in size decrease by the sixth millennium BC, and later a shift towards an onsite, millet-heavy diet during the Yangshao period after 4500 BC.

## Conclusions

The transition to millet agriculture in northern China comprised the coming together of a multitude of different subsistence elements that increased population growth and drove demic-diffusion at an initially greater rate than seen for rice populations to the south. This demographic growth saw the spread of millet agriculture beyond the regions of domestication centred on the Yellow River Basin (including the Wei Valley) and northeast China. The sparse amount of archaeobotanical and archaeozoological data makes reconstructing a timeline for domestication problematic and evidence is too limited to track the entire domestication trajectory from the beginnings of pre-domestication cultivation or pre-domestication pig management through to domestication for any specific region. However, grain size evidence suggests that pre-domestication cultivation began by at least 8300 BC (Zhao et al., 2020) with an acceleration in grain size seen for both foxtail and broomcorn millet between 6500 and 5500 BC, tied to the increased appearance of sedentary settlements. Dental morphometrics also support the domestication of pig during this same period in at least the middle Yellow River Basin starting by at least 6000–5500 BC. Unlike for rice, the fact that millet spikelet bases almost never survive archaeologically makes assessing the transition to non-shattering plants problematic. However, the appearance of harvesting knives around 4600–4300 BC might tentatively suggest that non-shattering varieties had become dominant by this period. As the domestication process unfolded, the process itself resulted in increased returns on the investment put into cultivation and herd management. This saw a growing number of calories obtained from millets to sustain human population growth and that of associated domestic pigs, which in turn made it increasingly difficult for millet-dependent communities to give up cultivation and return to foraging. The process of domesticating cereals in turn created labour traps (Fuller et al., 2010; Allaby et al., 2022) that required increasing investment in soil maintenance, harvesting and storage technologies.

Currently it appears that initial domestication processes for millets, and potentially pigs, was taking place in parallel in parts of the Yellow River region and central Loess Plateau (the Laoguantai of the Wei Valley, Peiligang, and Cishan regions) and in northeast China (the Liao River/Xinglongwa Culture region). The latter region has been hypothesised to have been associated with speakers of ancestral Transeurasian language speakers (Robbeets, 2017; Robbeets et al., 2020), while the former may be associated with early speakers of Sino-Tibetan (Sagart et al., 2019; Jacques & Stevens, 2024). Significantly, both the Dadiwan and Houli culture areas, in the west and east respectively, may also have initiated domestication of both millets and potentially pigs, although it is unclear if either of these regions witnessed a completed domestication process. It is probable that pre-domestication cultivation was begun but abandoned in some regions, such as the suggestion that the Dadiwan Culture may have been a ‘dead-end’ in terms of the *Panicum* domestication processes (Stevens & Fuller, 2017). In support of such theories it is notable that gaps in available settlement and radiocarbon data are evident for parts of northern China between ca. 5400 BC and 4500 BC (Leipe et al., 2019; Ren et al., 2021; Xu, D. et al., 2019), hence encompassing the Early Yangshao, Beixin and Zhaobagou Cultural Periods. It is likely, however, that we need to think in terms of a multicentric framework of agricultural origins across northern China, or a landscape perspective in which processes in different populations of crops were linked via gene flow facilitated by the exchange of grain that was then used as seed corn (Allaby et al., 2022). This constitutes a meta-population perspective on millets, in which genetic pools of cultivated crops form in distinct geographic regions, with gene flow through the movement of crops, or people with crops, between them, which incorporates earlier ideas on multicentric origins of domestication. Regardless, it is clear that millet domestication processes facilitated increased productivity and increased population density growth, which ultimately underpins the geographical expansion of migrating farmers. The isotopic evidence demonstrates that both pigs and dogs had been integrated as a fixed feature of human settlement sites by at least 4300–3600 BC, consuming a diet comprising high amounts of millets, transforming domesticated dogs into starch-eating agricultural village dogs. The switch from broomcorn to foxtail millet occurs around 4000 BC and probably marks a point at which the productivity of the enlarged denser panicle of foxtail millet had overtaken that of broomcorn millet. From 4000 to 3000 BC we see clearer evidence for increased grain size for both millets, possibly driven by increased deliberate human selection, perhaps supported by more intensive practices like manuring, and accompanied by further evidence for increased population size, seen in both settlement and radiocarbon data (Leipe et al., 2019; Ren et al., 2021). Taken together these trends supported an expanding population in terms of both density and size, while the limited productivity of millets meant that outward expansion in search of new land for cultivation would have been recurrent, accounting for the archaeological spread of specific Neolithic cultures, such as that of the Yangshao, as well as the Beixin and succeeding Dawenkou. It was this period of subsequent population growth that pushed populations of millet agriculturalists from around 3500 BC eastwards into the Korean Peninsula (Stevens et al., 2022) and Russian Primorye region (Sergusheva et al., 2022), west to edge of and onto the Tibetan Plateau (Dong et al., 2013; Jacques & Stevens, 2024) and southwards (c.3500–3000 BC) into southern China (Deng et al., 2022).

## Supporting information

Stevens et al. supplementary material 1 Stevens et al. supplementary material

Stevens et al. supplementary material 2 Stevens et al. supplementary material

Stevens et al. supplementary material 3 Stevens et al. supplementary material

## Data Availability

The data that contributes to the analyses and figures in this paper are provided in the supplementary materials.

## Supplementary material

The supplementary material for this article can be found at https://doi.org/10.1017/ehs.2024.31.

## Research transparency and reproducibility

All data are included in the supplementary materials of this article.
