# Supplementary material for: Millets, dogs, pigs and permanent settlement: productivity transitions in Neolithic northern China
Source: Evol Hum Sci. 2024 Nov 11;6:e44. doi: 10.1017/ehs.2024.31 (PMC11658956; doi:10.1017/ehs.2024.31)
Supplement: Stevens et al. supplementary material 1 — Stevens et al. supplementary material [file S2513843X24000318sup001.docx]

**Table S1. Representative larger sites through time, estimated median age and size. (plotted in Fig. 3).**

| **Site** | **Age** | **Size (hectares)** |
| --- | --- | --- |
| Xinglonggou | -5800 | 3 |
| Zhaobaogou | -4950 | 6 |
| Dongshanzui | -3485 |  |
| Fushanzhuang | -3500 | 35 |
| Xihe | -5900 | 10 |
| Dadiwan | -5500 | 1 |
| Wuluoxipo | -5250 | 2 |
| Bajuia | -5500 | 12 |
| Jiangzhai | -4600 | 5 |
| Banpo | -4500 | 5 |
| Diantoubao | -4000 | 70 |
| Cangdi | -4000 | 15.1 |
| Yangshaocun | -3750 | 30 |
| Xipo | -3600 | 40 |
| Dadiwan | -3200 | 50 |
| Dantu | -2800 | 13.4 |
| Liangchengzhen | -2200 | 272 |
| Taosi (Early) | -2200 | 56 |
| Yangwangcheng | -2200 | 367 |
| Zhangiazhaili | -2100 | 75 |
| Wangchenggang | -2000 | 50 |
| Taosi (Late) | -2000 | 289 |
| Taipu | -2000 | 70 |
| Shimao | -2000 | 400 |

**Table S2 Pre-Yangshao sites with archaeological millet evidence. (plotted in Fig. 4 - also in excel Table S3).**

| Site | Province | Latitude | Longitude | Broad Period | Early/ Late | Regional phase | Start Date BC/AD | Finish Date BC/AD | Est. Date Median BC/AD | wild Oryza | Panicum | Setaria | References |
| --- | --- | --- | --- | --- | --- | --- | --- | --- | --- | --- | --- | --- | --- |
| Bianbiandong | Shandong | 36.06167 | 118.4714 | Neolithic | - | Post-Houli | -5400 | -4900 | -5150 |  | Pa | Se | Sun et al. 2014 |
| Cishan | Hebei | 36.57585 | 114.1241 | Neolithic | Early | Cishan | -6102 | -5600 | -5851 |  | Pa | Se | Lu et al. 2009 |
| Dadiwan | Gansu | 35.00789 | 105.9089 | Neolithic | Early | Dadiwan | -5800 | -5400 | -5600 |  | Pa |  | Liu et al. 2004; Barton 2009 |
| Dingzhuang | Henan | 34.04989 | 113.8184 | Neolithic | Early | Peiligang | -7000 | -5000 | -6000 |  |  | Se | Song 2011 |
| Donghulin | Beijing | 39.987 | 115.7495 | Neolithic | Early | Doughulin | -9050 | -7550 | -8300 |  | Pa | Se | Yang et al. 2012; Zhao et al 2020 |
| Fudian | Henan | 34.56363 | 112.8648 | Neolithic | Early | Peiligang | -6000 | -5000 | -5500 |  |  | Se | Lee et al. 2007; Bestel et al. 2014 |
| Fuxin 12D16 | Inner Mongolia | 42.1557 | 121.896 | Neolithic | Early | Xinglongwa | -5500 | -5300 | -5400 |  | Pa | Se | Shelach-Lavi et al 2019 |
| Fuxin 12D56 | Inner Mongolia | 42.155 | 121.89 | Neolithic | Early | Xinglongwa | -5900 | -5700 | -5800 |  | Pa |  | Shelach-Lavi et al 2019 |
| Lixian 7 | Gansu | 34.18909 | 105.1784 | Neolithic | Early | Dadiwan I | -6050 | -5350 | -5700 |  | Pa |  | Ji, 2009; An et al. 2010 |
| Mangha | Inner Mongolia | 43.96963 | 122.1966 | Early Neolithic | (?) | (?) | - | - | -5800 |  | Pa | Se | Sun et al 2014 |
| Nanzhuangtou | Hebei | 39.11137 | 115.657 | Neolithic | Early | Nanzhuangtou | -8500 | -7700 | -8100 |  |  | Se | Yang et al. 2012; 2015 |
| Niuwabao | Hebei | 36.59404 | 114.0488 | Neolithic | Early | Cishan | -6500 | -5000 | -5750 |  |  | Se | Ren 1996; Liu et al 2008 |
| Peiligang | Henan | 34.43643 | 113.6583 | Neolithic | Early | Peiligang | -6043 | -5569 | -5806 |  |  | Se | Lu 1999: Table 4; Zhang and Hung 2013: Table 2 |
| Qianbuxia | Shandong | 36.74782 | 119.3816 | Neolithic | Early | Houli | -6500 | -5500 | -6000 |  | Pa | Se | Jin 2006 |
| Shawoli | Henan | 34.62597 | 113.6813 | Neolithic | Early | Peiligang | -5948 | -5692 | -5820 |  |  | Se | Wang 1984; Zhu 2013; Zhang and Hung 2010 |
| Shizitan Loc. 9 | Shanxi | 36.08992 | 110.5827 | Palaeolithic | Upper | - | -11850 | -10750 | -11300 |  |  | Se | Bestel et al. 2014 |
| Shizitan Loc. 9 | Shanxi | 36.08992 | 110.5827 | Palaeolithic | Upper | - | -10750 | -9650 | -10200 |  |  | Se | Bestel et al. 2014 |
| Wuluoxipo | Henan | 34.63843 | 113.0049 | Neolithic | Early | Peiligang | -6000 | -5000 | -5500 |  |  | Se | Zuo et al. 2016; Lee et al. 2007 |
| Xihe | Shandong | 36.70589 | 117.6301 | Neolithic | Early | Houli | -6070 | -5900 | -5985 | Or |  | Se | Jin et al 2014 |
| Xinglonggou | Inner Mongolia | 42.38727 | 120.0907 | Neolithic | Eary | Xinglongwa | -6200 | -5400 | -5800 |  | Pa | Se | Zhao 2004; Liu et al. 2015 |
| Xinle | Liaoning | 41.848 | 123.4137 | Neolithic | Early | Xinle | -5500 | -4500 | -5000 |  | Pa |  | Yan 1992 |
| Yuezhuang | Shandong | 36.61994 | 116.8286 | Neolithic | Early | Houli | -6060 | -5750 | -5905 | Or | Pa | Se | Crawford et al.2006 |
| Zhangmatun | Shandong | 36.71993 | 117.1128 | Neolithic | Early | - | -7050 | -6500 | -6775 |  | Pa | Se | Wu et al. 2014 |
| Zhuzhai | Henan | 34.82523 | 113.3055 | Neolithic | Early | - | -5974 | -5823 | -5898.5 | Or | Pa | Se | Bestel et al 2018 |

**Table S3. Millet sites in China, Korea, Japan, and Russia (plotted in Fig. 5. see separate csv/excel file).**

**Table S4. Grain metrics for archaeological *Panicum* and *Setaria*. (plotted in Fig. 6. see separate csv / excel file).**

**Table S5. Isotopic measurements on Chinese pig or dog collagen. (plotted in Fig. 7 and Fig. 8 bottom)**

| **Species** | **Site** | **Culture** | **Age BC** | **δ^13^C** | **δ ^15^N** | **source** |
| --- | --- | --- | --- | --- | --- | --- |
| dog | Xinglonggou 1 | Xinlongwa | -5800 | -11 | 8.5 | Liu et al 2012 |
| dog | Xinglonggou 1 | Xinlongwa | -5800 | -11.6 | 9.4 | Liu et al 2012 |
| dog | Xinglonggou 1 | Xinlongwa | -5800 | -19 | 8.3 | Liu et al 2012 |
| dog | Xinglonggou 1 | Xinlongwa | -5800 | -17.2 | 8.1 | Liu et al 2012 |
| dog | Xinglonggou 1 | Xinlongwa | -5800 | -11.9 | 8.3 | Liu et al 2012 |
| dog | Xinglonggou 1 | Xinlongwa | -5800 | -21.2 | 6.1 | Liu et al 2012 |
| dog | Dadiwan | Dadiwan | -5500 | -19.9 | 6.2 | Barton et al. 2009 |
| dog | Dadiwan | Dadiwan | -5500 | -19.8 | 5.9 | Barton et al. 2009 |
| dog | Dadiwan | Dadiwan | -5500 | -10.2 | 7.3 | Barton et al. 2009 |
| dog | Dadiwan | Dadiwan | -5500 | -10.2 | 7.5 | Barton et al. 2009 |
| dog | Dadiwan | Dadiwan | -5500 | -11.1 | 7.7 | Barton et al. 2009 |
| Dog | Wayaogou | Banpo | -4300 | -11 | 9.7 | Chen et al 2016 |
| dog | Wayaogou | Banpo | -4300 | -10.9 | 7.1 | Chen et al 2016 |
| dog | Wayaogou | Banpo | -4300 | -11.3 | 9.7 | Chen et al 2016 |
| dog | Xipo | Yangshao | -3800 | -8.18 | 6.91 | Pechenkina et al. 2005 |
| dog | Dadiwan | Late Banpo | -3750 | -7.9 | 7.9 | Barton et al. 2009 |
| dog | Dadiwan | Late Banpo | -3750 | -8.2 | 8.6 | Barton et al. 2009 |
| dog | Dadiwan | Late Banpo | -3750 | -10.7 | 8.7 | Barton et al. 2009 |
| dog | Dadiwan | Late Banpo | -3750 | -9.3 | 9 | Barton et al. 2009 |
| dog | Dadiwan | Late Banpo | -3750 | -13 | 8.6 | Barton et al. 2009 |
| dog | Dadiwan | Late Banpo | -3750 | -13.1 | 8.7 | Barton et al. 2009 |
| Dog | Dongying | Late Yangshao | -2550 | -14.6 | 6.9 | Chen et al 2016 |
| dog | Dongying | Late Yangshao | -2550 | -9.1 | 5.9 | Pechenkina et al. 2005 |
| dog | Kangjia | Longshan | -2300 | -8.97 | 9.48 | Pechenkina et al. 2005 |
| dog | Kangjia | Longshan | -2300 | -14.53 | 9.84 | Pechenkina et al. 2005 |
| dog | Wadian | Late Longshan | -2100 | -10.9 | 8.1 | Chen et al. 2016b |
| dog | Wadian | Late Longshan | -2100 | -10.5 | 5.9 | Chen et al. 2016b |
| dog | Wadian | Late Longshan | -2100 | -11 | 6.1 | Chen et al. 2016b |
| dog | Wadian | Late Longshan | -2100 | -10.6 | 7.2 | Chen et al. 2016b |
| dog | Wadian | Late Longshan | -2100 | -9.1 | 6.5 | Chen et al. 2016b |
| dog | Wadian | Late Longshan | -2100 | -10.3 | 8.5 | Chen et al. 2016b |
| dog | Wadian | Late Longshan | -2100 | -8.5 | 8.4 | Chen et al. 2016b |
| dog | Xinglonggou III | Lower Xiajiadian | -1900 | -7.4 | 7.1 | Liu et al 2012 |
| dog | Zhangdeng | Proto-Shang | -1700 | -7.6 | 7.6 | Hou et al. 2013 |
| dog | Zhangdeng | Proto-Shang | -1700 | -8.8 | 6.7 | Hou et al. 2013 |
| dog | Zhangdeng | Proto-Shang | -1700 | -6.7 | 7.3 | Hou et al. 2013 |
| dog | Zhangdeng | Proto-Shang | -1700 | -7.4 | 7.7 | Hou et al. 2013 |
| **Species** | **Site** | **Culture** | **Age BC** | **δ^13^C** | **δ ^15^N** | **source** |
| pig | Yuezhuang | Houli | -5905 | -18.1 | 4.7 | Hu et al 2008 |
| pig | Yuezhuang | Houli | -5905 | -19 | 9.1 | Hu et al 2008 |
| pig | Yuezhuang | Houli | -5905 | -10.6 | 6.4 | Hu et al 2008 |
| pig | Yuezhuang | Houli | -5905 | -20 | 6 | Hu et al 2008 |
| pig | Xinlonggou 1 | Xinlongwa | -5800 | -20.9 | 4.8 | Liu et al 2012 |
| pig | Xinlonggou 1 | Xinlongwa | -5800 | -20.3 | 4.8 | Liu et al 2012 |
| pig | Xinlonggou 1 | Xinlongwa | -5800 | -19.6 | 4.5 | Liu et al 2012 |
| pig | Xinlonggou 1 | Xinlongwa | -5800 | -20.6 | 5.3 | Liu et al 2012 |
| pig | Xinlonggou 1 | Xinlongwa | -5800 | -22 | 3.2 | Liu et al 2012 |
| pig | Xinlonggou 1 | Xinlongwa | -5800 | -18.3 | 7.2 | Liu et al 2012 |
| pig | Xinlonggou 1 | Xinlongwa | -5800 | -20.3 | 4.6 | Liu et al 2012 |
| pig | Xinlonggou 1 | Xinlongwa | -5800 | -20 | 5.1 | Liu et al 2012 |
| pig | Xinlonggou 1 | Xinlongwa | -5800 | -20 | 5.1 | Liu et al 2012 |
| pig | Xinlonggou 1 | Xinlongwa | -5800 | -20.2 | 4.6 | Liu et al 2012 |
| pig | Xinlonggou 1 | Xinlongwa | -5800 | -19.9 | 4 | Liu et al 2012 |
| pig | Xinlonggou 1 | Xinlongwa | -5800 | -21.6 | 2.8 | Liu et al 2012 |
| pig | Xinlonggou 1 | Xinlongwa | -5800 | -19.8 | 5 | Liu et al 2012 |
| pig | Dadiwan | Dadiwan | -5500 | -12 | 8.3 | Barton et al. 2009 |
| pig | Dadiwan | Dadiwan | -5500 | -16.3 | 6.2 | Barton et al. 2009 |
| pig | Dadiwan | Dadiwan | -5500 | -20.4 | 5.6 | Barton et al. 2009 |
| pig | Dadiwan | Dadiwan | -5500 | -20.9 | 7.2 | Barton et al. 2009 |
| pig | Dadiwan | Dadiwan | -5500 | -19.3 | 5.3 | Barton et al. 2009 |
| pig | Dadiwan | Dadiwan | -5500 | -19.1 | 7 | Barton et al. 2009 |
| pig | Dadiwan | Dadiwan | -5500 | -19 | 5.6 | Barton et al. 2009 |
| Pig | Wayaogou | Banpo | -4300 | -16.6 | 7.2 | Chen et al 2016a |
| Pig | Wayaogou | Banpo | -4300 | -10.7 | 5.9 | Chen et al 2016a |
| Pig | Wayaogou | Banpo | -4300 | -17.6 | 4.7 | Chen et al 2016a |
| Pig | Wayaogou | Banpo | -4300 | -17 | 5.4 | Chen et al 2016a |
| Pig | Wayaogou | Banpo | -4300 | -9 | 6.3 | Chen et al 2016a |
| Pig | Wayaogou | Banpo | -4300 | -10.9 | 6.4 | Chen et al 2016a |
| Pig | Wayaogou | Banpo | -4300 | -10.1 | 6.2 | Chen et al 2016a |
| Pig | Wayaogou | Banpo | -4300 | -12.4 | 5 | Chen et al 2016a |
| pig | Xipo | Yangshao | -3800 | -7.4 | 7.49 | Pechenkina et al. 2005 |
| pig | Xipo | Yangshao | -3800 | -7.65 | 7.96 | Pechenkina et al. 2005 |
| Pig | Dongying | Yangshao | -3750 | -9.8 | 6.1 | Chen et al 2016 |
| Pig | Dongying | Yangshao | -3750 | -9.2 | 7.7 | Chen et al 2016 |
| pig | Dadiwan | Late Banpo | -3750 | -8.5 | 8 | Barton et al. 2009 |
| pig | Dadiwan | Late Banpo | -3750 | -15.7 | 7.2 | Barton et al. 2009 |
| pig | Dadiwan | Late Banpo | -3750 | -8.3 | 8.4 | Barton et al. 2009 |
| pig | Dadiwan | Late Banpo | -3750 | -14.7 | 8.5 | Barton et al. 2009 |
| pig | Dadiwan | Late Banpo | -3750 | -17.5 | 6.4 | Barton et al. 2009 |
| pig | Dadiwan | Late Banpo | -3750 | -7 | 7.7 | Barton et al. 2009 |
| pig | Dadiwan | Late Banpo | -3750 | -9.7 | 9.2 | Barton et al. 2009 |
| pig | Dadiwan | Late Banpo | -3750 | -9 | 9.2 | Barton et al. 2009 |
| pig | Dadiwan | Late Banpo | -3750 | -10 | 8.8 | Barton et al. 2009 |
| pig | Dadiwan | Late Banpo | -3750 | -6.3 | 8.1 | Barton et al. 2009 |
| pig | Dadiwan | Late Banpo | -3750 | -6.5 | 8.6 | Barton et al. 2009 |
| pig | Dadiwan | Late Banpo | -3750 | -8.8 | 8.4 | Barton et al. 2009 |
| pig | Dadiwan | Late Banpo | -3750 | -11.5 | 9.9 | Barton et al. 2009 |
| pig | Dadiwan | Late Banpo | -3750 | -12.2 | 8.9 | Barton et al. 2009 |
| pig | Dadiwan | Late Banpo | -3750 | -9.1 | 9.1 | Barton et al. 2009 |
| pig | Dadiwan | Late Banpo | -3750 | -11.4 | 9.6 | Barton et al. 2009 |
| pig | Dadiwan | Late Banpo | -3750 | -9.2 | 8.8 | Barton et al. 2009 |
| pig | Dadiwan | Late Banpo | -3750 | -8.2 | 9.1 | Barton et al. 2009 |
| pig | Dadiwan | Late Banpo | -3750 | -8.3 | 8.7 | Barton et al. 2009 |
| pig | Dadiwan | Late Banpo | -3750 | -14.9 | 6.8 | Barton et al. 2009 |
| pig | Dadiwan | Late Banpo | -3750 | -9 | 8.4 | Barton et al. 2009 |
| pig | Dadiwan | Late Banpo | -3750 | -9 | 8.6 | Barton et al. 2009 |
| pig | Dadiwan | Late Banpo | -3750 | -7.7 | 7.9 | Barton et al. 2009 |
| pig | Dadiwan | Late Banpo | -3750 | -8.3 | 8.9 | Barton et al. 2009 |
| pig | Dadiwan | Late Banpo | -3750 | -9 | 8.2 | Barton et al. 2009 |
| pig | Dadiwan | Late Banpo | -3750 | -11 | 7.8 | Barton et al. 2009 |
| pig | Dadiwan | Late Banpo | -3750 | -12.3 | 9.3 | Barton et al. 2009 |
| pig | Dadiwan | Late Banpo | -3750 | -8.3 | 7.6 | Barton et al. 2009 |
| pig | Xinglonggou II | Hongshan | -3750 | -19.8 | 3.8 | Liu et al 2012 |
| pig | Xinglonggou II | Hongshan | -3750 | -20.9 | 3.4 | Liu et al 2012 |
| Pig | Dongying | Late Yangshao | -2550 | -14.6 | 6.3 | Chen et al 2016a |
| Pig | Dongying | Late Yangshao | -2550 | -15.4 | 5.9 | Chen et al 2016a |
| Pig | Dongying | Late Yangshao | -2550 | -8 | 7.5 | Chen et al 2016a |
| Pig | Dongying | Late Yangshao | -2550 | -7.8 | 9.1 | Chen et al 2016a |
| Pig | Dongying | Late Yangshao | -2550 | -8.2 | 7.6 | Chen et al 2016a |
| Pig | Dongying | Late Yangshao | -2550 | -9.5 | 7.1 | Chen et al 2016a |
| Pig | Dongying | Late Yangshao | -2550 | -9.7 | 7.4 | Chen et al 2016a |
| pig | Kangjia | Longshan | -2300 | -11.53 | 7.77 | Pechenkina et al. 2005 |
| pig | Kangjia | Longshan | -2300 | -11.76 | 9.57 | Pechenkina et al. 2005 |
| pig | Kangjia | Longshan | -2300 | -7.53 | 8.71 | Pechenkina et al. 2005 |
| pig | Wadian | Late Longshan | -2100 | -12.3 | -12.4 | Chen et al. 2016b |
| pig | Wadian | Late Longshan | -2100 | -27.7 |  | Chen et al. 2016b |
| pig | Wadian | Late Longshan | -2100 | -11.8 | 5.5 | Chen et al. 2016b |
| pig | Wadian | Late Longshan | -2100 | -12.4 | 7.5 | Chen et al. 2016b |
| pig | Wadian | Late Longshan | -2100 | -24.2 |  | Chen et al. 2016b |
| pig | Wadian | Late Longshan | -2100 | -11.3 | 5.7 | Chen et al. 2016b |
| pig | Wadian | Late Longshan | -2100 | -13.2 | 6.1 | Chen et al. 2016b |
| pig | Wadian | Late Longshan | -2100 | -11.3 | 7.2 | Chen et al. 2016b |
| pig | Wadian | Late Longshan | -2100 | -16.1 | 8.7 | Chen et al. 2016b |
| pig | Wadian | Late Longshan | -2100 | -8.1 | 6.9 | Chen et al. 2016b |
| pig | Wadian | Late Longshan | -2100 | -8.3 | 6.8 | Chen et al. 2016b |
| pig | Wadian | Late Longshan | -2100 | -9.2 | 7.7 | Chen et al. 2016b |
| pig | Xinglonggou III | Lower Xiajiadian | -1900 | -6.8 | 8.8 | Liu et al 2012 |
| pig | Xinglonggou III | Lower Xiajiadian | -1900 | -8.1 | 9 | Liu et al 2012 |
| pig | Xinglonggou III | Lower Xiajiadian | -1900 | -7.7 | 7.1 | Liu et al 2012 |
| pig | Xinglonggou III | Lower Xiajiadian | -1900 | -19.3 | 3.9 | Liu et al 2012 |
| pig | Xinglonggou III | Lower Xiajiadian | -1900 | -7.1 | 8.5 | Liu et al 2012 |
| pig | Xinglonggou III | Lower Xiajiadian | -1900 | -13.3 | 7.2 | Liu et al 2012 |
| pig | Xinglonggou III | Lower Xiajiadian | -1900 | -11.3 | 7 | Liu et al 2012 |
| pig | Xinglonggou III | Lower Xiajiadian | -1900 | -7.4 | 8.4 | Liu et al 2012 |
| pig | Zhangdeng | Proto-Shang | -1700 | -6.5 | 7.7 | Hou et al. 2013 |
| pig | Zhangdeng | Proto-Shang | -1700 | -8.8 | 7.1 | Hou et al. 2013 |
| pig | Zhangdeng | Proto-Shang | -1700 | -7.1 | 7.6 | Hou et al. 2013 |
| pig | Zhangdeng | Proto-Shang | -1700 | -9.2 | 7.8 | Hou et al. 2013 |
| pig | Zhangdeng | Proto-Shang | -1700 | -6.9 | 7.1 | Hou et al. 2013 |
| pig | Zhangdeng | Proto-Shang | -1700 | -11.2 | 7.9 | Hou et al. 2013 |
| pig | Zhangdeng | Proto-Shang | -1700 | -6.8 | 7.5 | Hou et al. 2013 |
| pig | Zhangdeng | Proto-Shang | -1700 | -9.1 | 7.7 | Hou et al. 2013 |
| pig | Zhangdeng | Proto-Shang | -1700 | -6.5 | 8.1 | Hou et al. 2013 |
| pig | Zhangdeng | Proto-Shang | -1700 | -6.4 | 7.4 | Hou et al. 2013 |
| pig | Zhangdeng | Proto-Shang | -1700 | -8.8 | 8 | Hou et al. 2013 |
| pig | Zhangdeng | Proto-Shang | -1700 | -8 | 6.6 | Hou et al. 2013 |
| pig | Zhangdeng | Proto-Shang | -1700 | -6.6 | 8.4 | Hou et al. 2013 |
| pig | Zhangdeng | Proto-Shang | -1700 | -6.9 | 8.3 | Hou et al. 2013 |
| pig | Zhangdeng | Proto-Shang | -1700 | -7.5 | 8.2 | Hou et al. 2013 |
| pig | Zhangdeng | Proto-Shang | -1700 | -6.4 | 7.6 | Hou et al. 2013 |
| pig | Zhangdeng | Proto-Shang | -1700 | -7.3 | 7.1 | Hou et al. 2013 |
| pig | Zhangdeng | Proto-Shang | -1700 | -8.1 | 7.6 | Hou et al. 2013 |
| pig | Zhangdeng | Proto-Shang | -1700 | -7.3 | 7.8 | Hou et al. 2013 |

**Table S6. Archaeological Pig metrics (M_3_). (Plotted in Fig. 8 top and middle; data mainly from Luo 2012; Wang et al. 2015; plus Song et al. 2019).**

| **Regions** | **Lattitude** | **Longitude** | **Site** | **Period** | **n** | **Median Age (BC)** | **Maximum** | **Minimum** | **Average** | **Standard deviation** |
| --- | --- | --- | --- | --- | --- | --- | --- | --- | --- | --- |
| Huai | 33.62003 | 113.67398 | Jiahu | Peiligang | 12 | -6200 | 46.66 | 36.39 | 42.23 | 3.074 |
| Huai | 33.850178 | 116.81665 | Shishanzi | Shishanzi Culture | 3 | -4900 | 40 | 36 | 38.33 | 2.082 |
| Huai | 31.426149 | 119.60866 | Xixi | Majiabang | 2 | -4450 | 38.06 | 37.99 | 38.03 | 0.0620358 |
| Huai | 32.84267 | 119.50378 | Longqiuzhuang | Longqiuzhuang | 5 | -4250 | 42.3 | 32.4 | 38.72 | 2.127 |
| Huai | 31.687743 | 119.49164 | Sanxingcun | Sanxingcun | 16 | -4000 | 41.08 | 34.08 | 37.07 | 2.692 |
| Huai | 33.358139 | 116.74968 | Yuchisi | Late Dawenkou | 12 | -2700 | 39.5 | 30.1 | 36.19 | 3.722 |
| Huai | 33.358139 | 116.74968 | Yuchisi | Longshan | 11 | -2350 | 44.6 | 28.18 | 34.12 | 3.332 |
| Huai | 32.349686 | 118.35082 | Heying | L. Shang/ Western Zhou | 11 | -1000 | 38.68 | 28.74 | 32.96 | 3.748 |
| Northeast | 42.387265 | 120.0907 | Xinglonggou | Xinglongwa | 24 | -5600 | 49.74 | 32.16 | 42.35 | 3.535 |
| Northeast | 43.307245 | 118.24077 | Baiyinchanghan | Xinglongwa | 3 | -5550 | 46.6 | 41.5 | 43.4 | 2.787 |
| Northeast | 42.388145 | 120.12193 | Xinglongwa | Xinglongwa | 74 | -5500 | 49.27 | 32.87 | 42.32 | 3.447 |
| Northeast | 42.352249 | 120.16786 | Zhaobaogou | Zhaobaogou | 3 | -4850 | 45 | 41 | 43.3 | 2.066 |
| Northeast | 42.330121 | 120.69642 | Dadianzi | Lower Xiajiadian Culture | 3 | -1600 | 39.38 | 36 | 37.17 | 1.915 |
| Northeast | 39.07985 | 121.70023 | Dazuizi | Shang period | 4 | -1300 | 37 | 30.5 | 32.75 | 2.958 |
| Yangtze | 25.214385 | 110.28437 | Zengpiyan | 12000-7000 BP | 12 | -9500 | 47.46 | 36.57 | 41.31 | 3.632 |
| Yangtze | 30.037222 | 107.86056 | Lower Yuxi | Chengbeixi Culture (8500-700 BP) | 2 | -5100 | 36.3 | 35.5 | 35.9 | 0.565 |
| Yangtze | 25.020501 | 99.035929 | Tangzigou | Tangzigou Culture | 4 | -5000 | 47 | 35.83 | 41.48 | 4.746 |
| Yangtze | 30.891489 | 110.883 | Liulinxi | Chengbeixi Culture | 2 | -4950 | 41.8 | 41.6 | 41.7 | 0.141 |
| Yangtze | 30.14459 | 120.21678 | Kuahuqiao | Kuahuqiao Culture (8000-7000 BC) | 13 | -5500 | 42.37 | 34.29 | 38.54 | 2.942 |
| Yangtze | 30.4032 | 110.61633 | Shazui | Daxi Culture (7000-5300 BP) | 10 | -4150 | 44.54 | 29 | 38.7 | 5.657 |
| Yangtze | 31.718677 | 120.06586 | Weidun | Majiabang (7000-5500 BP) | 11 | -4050 | 47.5 | 36.5 | 40.7 | 3.4668928 |
| Yangtze | 23.886187 | 106.54818 | Gexinqiao | c.6000 BP | 3 | -4000 | 39.4 | 36.7 | 38.63 | 1.686 |
| Yangtze | 29.869105 | 116.03475 | Saidun | Xuejiagang Culture (5500-4800 BP) | 13 | -3100 | 47 | 30 | 37.42 | 3.92 |
| Yangtze | 32.273717 | 112.94924 | Diaolongbei Phase 3 | 5300-4800 BP | 16 | -3050 | 39.35 | 33.19 | 36.03 | 2.165 |
| Yangtze | 31.15817 | 121.4607 | Maqiao | Liangzhu Culture | 1 | -2300 |  |  | 38.08 |  |
| Yangtze | 31.15817 | 121.4607 | Maqiao | Maqiao Culture | 9 | -1600 | 42.94 | 36.64 | 40.15 | 2.184 |
| Yellow Riv | 36.61994 | 116.82861 | Yuezhuang | Houli (Pre-Yangshao) |  | -6100 |  |  | 43.55 |  |
| Yellow Riv | 36.575851 | 114.12407 | Cishan | Cishan (Pre-Yangshao) Culture | 3 | -6000 | 45 | 39.2 | 41.4 | 3.143 |
| Yellow Riv | 36.705893 | 117.63012 | Xihe | Houli (Pre-Yangshao) | 3 | -5800 | 48.44 | 35.68 | 39.95 | 7.5388315 |
| Yellow Riv | 35.00789 | 105.90893 | Dadiwan | Laoguantai (Pre-Yangshao) Culture to Late Yangshao Period | 66 | -5500 | 46 | 30.8 | 37.06 | 3.2267309 |
| Yellow Riv | 34.274517 | 109.048 | Banpo | Early Yangshao Culture | 1 | -4900 |  |  | 35.8 |  |
| Yellow Riv | 35.650252 | 111.31338 | Zhucun | Early Yangshao Culture | 1 | -4700 |  |  | 36.6 |  |
| Yellow Riv | 40.527477 | 112.7195 | Shihushan I | Early Yangshao Culture | 7 | -4600 | 42.7 | 35.4 | 39.41 | 2.573 |
| Yellow Riv | 36.74782 | 119.38156 | Qianbuxia | Houli (Pre-Yangshao) Culture to Dawenkou Culture | 2 | -4550 | 39.8 | 36.3 | 38.05 | 2.475 |
| Yellow Riv | 35.00088 | 109.03325 | Wayaogou | 7000-6000 BP | 18 | -4500 | 43.5 | 33.5 | 39.1 | 2.7472075 |
| Yellow Riv | 35.708568 | 114.99935 | Xishuipo | Early Yangshao Culture | 196 | -4250 | 47.28 | 30.06 | 37.8 | 2.978 |
| Yellow Riv | 34.386072 | 107.15504 | Beishouling | Late Laoguantai (Pre-Yangshao) Culture to Early Yangshao Culture | 25 | -4000 | 44 | 32 | 37.99 | 2.749 |
| Yellow Riv | 34.535052 | 109.8632 | Quanhucun | Middle Yangshao Culture | 18 | -3750 | 44.7 | 28.4 | 33.5 | 4.4779482 |
| Yellow Riv | 34.498614 | 110.70213 | Xipo | Middle Yangshao Culture | 21 | -3100 | 39.27 | 27.1 | 34.67 | 3.025 |
| Yellow Riv | 34.915174 | 113.54054 | Xishan | Late Yangshao Culture | 5 | -3050 | 39 | 29 | 35.4 | 4.099 |
| Yellow Riv | 34.378741 | 109.21794 | Jiangzhai | Early Yangshao Culture to Longshan Culture | 19 | -3000 | 41 | 31.4 | 36.24 | 2.127 |
| Yellow Riv | 34.620925 | 110.32183 | Xiwangcun | Late Yangshao Culture | 3 | -2975 | 39.9 | 32.31 | 35.31 | 4.037 |
| Yellow Riv | 34.506689 | 117.79506 | Liangwangcheng | Dawenkou | 2 | -2750 | 37.26 | 33.46 | 36.36 | 3.3676598 |
| Yellow Riv | 34.930211 | 117.41169 | Jianxin | Late Dawenkou (5000-4500 BP) | 4 | -2700 | 36.65 | 30.38 | 33.4 | 3.0455373 |
| Yellow Riv | 34.955829 | 112.28364 | Zhouli III | Miaodigou II Period | 1 | -2400 | 36.29 |  | 36.29 |  |
| Yellow Riv | 36.409345 | 116.26095 | Jiaochangpu | Longshan Culture | 7 | -2200 | 37.09 | 31.19 | 34.7 | 2.361 |
| Yellow Riv | 34.187452 | 113.40494 | Wadian | Longshan Culture | 5 | -2150 | 37.49 | 30.4 | 35.13 | 2.804 |
| Yellow Riv | 35.67515 | 111.40137 | Taosi | Longshan Culture | 5 | -2125 | 36.09 | 29.83 | 33.02 | 2.373 |
| Yellow Riv | 34.11667 | 115.18333 | Shantaisi | Longshan Culture | 14 | -2100 | 37.31 | 29.28 | 33.2 | 2.591 |
| Yellow Riv | 34.473131 | 113.85836 | Guchengzhai | Erlitou Culture | 4 | -1850 | 35.22 | 29.04 | 32.1 | 3.187 |
| Yellow Riv | 33.636378 | 109.98016 | Donglongshan | End of Neolithic to Early Bronze Age | 11 | -1825 | 37.2 | 24.7 | 31.7 | 3.9396509 |
| Yellow Riv | 34.70329 | 112.71664 | Erlitou | Erlitou Culture | 21 | -1800 | 36.78 | 27.3 | 33.45 | 2.673 |
| Yellow Riv | 39.644814 | 110.43185 | Zhukaigou | Late Longshan Period to Early Shang Period | | -1600 | 36.5 | 29.1 | 32.8 |  |
| Yellow Riv | 34.473131 | 113.85836 | Guchengzhai | Early Shang | 4 | -1550 | 31.47 | 27.06 | 29.39 | 1.834 |
| Yellow Riv | 34.726725 | 112.7668 | Yanshi | Early Shang | 468 | -1500 | 39.82 | 23.44 | 32.7 | 2.899 |
| Yellow Riv | 34.70329 | 112.71664 | Erlitou | Early Shang | 9 | -1500 | 36.8 | 30.06 | 33.39 | 2.478 |
| Yellow Riv | 36.14148 | 114.33711 | Huayuanzhuang | Middle Shang | 8 | -1300 | 37.7 | 27.08 | 31.42 | 3.311 |
| Yellow Riv | 36.120856 | 114.32416 | Yinxu | Late Shang |  | -1275 |  |  | 31.4 |  |
| Yellow Riv | 36.113782 | 114.31332 | Heihelu | Late Shang | 98 | -1250 | 39.08 | 26.23 | 32.96 | 2.696 |
| Yellow Riv | 37.414487 | 110.78855 | Gaohong | Shang Period | 2 | -1200 | 32.21 | 28.83 | 30.52 | 2.39 |
| Yellow Riv | 34.473131 | 113.85836 | Guchengzhai | Late Shang | 6 | -1200 | 32.19 | 24.91 | 28.56 | 2.574 |
| Yellow Riv | 34.898975 | 117.22149 | Qianzhangda | Late Shang to Early Western Zhou | 40 | -1150 | 39.62 | 23.33 | 32.54 | 3.108 |
| Yellow Riv | 34.212782 | 108.72212 | Fengxi | Late Shang to Western Zhou | 5 | -1050 | 29.8 | 26.43 | 28.15 | 2.356 |
| Yellow Riv | 35.735798 | 111.56082 | Tianma-Qucun | Western Zhou | 29 | -900 | 36 | 26 | 31.59 | 2.4648512 |
| Yellow Riv | 34.396971 | 113.74747 | Zhonghang | Spring and Autumn | 4 | -650 | 32.85 | 25.35 | 30.1 | 3.6429872 |
| Yellow Riv | 35.735798 | 111.56082 | Tianma-Qucun | Spring and Autumn | 10 | -600 | 37 | 27 | 31.27 | 3.2493802 |
| Yellow Riv | 34.898975 | 117.22149 | Qianzhangda | Eastern Zhou | 3 | -500 | 35.93 | 28.5 | 32.23 | 3.715 |
| Yellow Riv | 34.396971 | 113.74747 | Zhonghang | Warring States | 28 | -400 | 38.7 | 25.4 | 30.93 | 2.681 |
| Yellow Riv | 34.473131 | 113.85836 | Guchengzhai | Warring States | 2 | -300 | 34.69 | 30.51 | 32.6 | 2.956 |

**References cited in Supplementary materials above.**

An CB, Ji Duxue, Chen FH, et al.

2010 Evolution of prehistoric agriculture in central Gansu Province, China: A case study in Qin’an and Li County. Chinese Science Bulletin 55: 1925-1930.

Archaeology, Shaanxi Provincial Institute of

2004 Lintong Lingkoucun. Xi'an: Sanqin Press.

Barton, Loukas. 2009. *Early Food Production in China’s Western Loess Plateau*. PhD Dissertation, University of California, Davis.

Bestel, Sheahan, Gary W Crawford, Li Liu, Jinming Shi, Yanhua Song and Xingcan Chen.

2014. The Evolution of Millet Domestication, Middle Yellow River Region, North China: Evidence from Charred Seeds at the Late Palaeolithic Shizitan S9 Site. *The Holocene* 24(3): 261‐265.

Bestel, S., Bao, Y., Zhong, H., Chen, X. and Liu, L.

2018. Wild plant use and multi-cropping at the early Neolithic Zhuzhai site in the middle Yellow River region, China. *The Holocene*, *28*(2), pp.195-207.

Chen XX.

2007 Analysis of flotation soil sample results from two sites in Rizhao, Shandong (in Chinese with English abstract). Nanfang Wenwu (Southern Cultural Relics) 2007(1): 92-94.

Chen, X. L., Hu, S. M., Hu, Y. W., Wang, W. L., Ma, Y. Y., Lü, P., & Wang, C. S.

2016a. Raising practices of Neolithic livestock evidenced by stable isotope analysis in the Wei River valley, North China. International Journal of Osteoarchaeology, 26(1), 42-52.

Chen, X. L., Fang, Y. M., Hu, Y. W., Hou, Y. F., Lü, P., Yuan, J., ... & Richards, M. P.

2016b. Isotopic reconstruction of the Late Longshan Period (ca. 4200–3900 BP) dietary complexity before the onset of state‐level societies at the Wadian site in the Ying River Valley, Central Plains, China. International Journal of Osteoarchaeology, 26(5), 808-817.

Cheng ZJ, Yang YZ, Zhang JZ, Yu J, Chen BB, Zhang H and Gong XC

2016 A study of charred plant remains from the Yangbao [Yangpu] site of Suzhou, Anhui (in Chinese). Jianghan Kaogu (Jianghan Archaeology) 142(1): 95-103

Crawford, Gary W, Chen XX, and Wang JH.

2006. Carbonised rice from the Houli Culture site of Yuezhuang, Changqing, Jinan, Shandong (in Chinese). *Dongfang Kaogu (Oriental Archaeology)* 3: 247–251.

Deng ZH, Qing L, Gao Y et al

2015 From early domesticated rice of the Middle Yangtze basin to millet, rice and wheat agriculture: Archaeobotanical macro-remains from Baligang, Nanyang basin, Central China (6700–500bc). PLoS ONE 10:e0139885

Hou, L., Hu, Y., Zhao, X., Li, S., Wei, D., Hou, Y., ... & Wang, C.

2013. Human subsistence strategy at Liuzhuang site, Henan, China during the proto-Shang culture (∼ 2000–1600 BC) by stable isotopic analysis. *Journal of Archaeological Science*, *40*(5), 2344-2351.

Hu, Y., Wang, S., Luan, F., Wang, C., & Richards, M. P.

2008. Stable isotope analysis of humans from Xiaojingshan site: implications for understanding the origin of millet agriculture in China. *Journal of archaeological Science*, *35*(11), 2960-2965.

Gao S, Sun ZY, Shao J, Wei X, Zhao ZJ

2016 The analysis of the flotation results from the Zhaimaoliang site, in Yulin, Shaanxi, Shaanxi (in Chinese). Agricultural Archaeology 2016(3): 14-19

Gu, D.

2003 The Formation Processes and Environmental Degradations of Lagoonal Wetlands along Shandong Peninsula: A Case Study of Chaoyanggang Lagoon (in Chinese with English abstract), Ocean University of China.

Ji, DX

2009 Possible Causes of the Transition from Collecting Economy to Agricultural Economy in Northwest China (in Chinese). *Kaogu yu Wenwu* (*Archaeology and Cultural Relics)* 2009 (4): 36-47

Jin, G.Y.

2006 Phytolith analysis for the archaeological sites of pre-Qin dynasties in Shandong and some related questions. Dongfang Kaogu [Oriental Archaeology] 3:258-279.

Jin, G.Y. Wu WW, Zhang KS et al.

2014 8000‐Year old rice remains from the north edge of the Shandong Highlands, East China. Journal of Archaeological Science 51: 34‐42.

Kong, ZC, Liu CJ, He DL

1999 Plant remains from the Zhuanglixi Site in Tengzhou City, Shandong and their significance to environmental archaeology (in Chinese). Kaogu (Archaeology) 1999(7): 69-64.

Lee, Gyoung‐Ah, Gary W Crawford, Li Liu Li and Xingcan Chen.

2007. Plants and people from the early Neolithic to the Shang periods in North China. *Proceedings of the National Academy of Sciences* 104(3): 1087‐1092.

Liu, C.J., Jin, G.Y. and Kong, Z.C.

2008 Archaeobotany: Research on seeds and fruits. Beijing: Science Press.

Liu CZ, Kong ZC and Lang SD

2004 Plant remains at the Dadiwan site and a discussion of human adaptation to the environment (in Chinese). *Zhongyuan Wenwu* (Cultural Relics of Central China) 2004(4): 26-30.

Liu, G.X.

2006 Retrospect and rethinking on the excavation of location one at the Xinglonggou site (in Chinese). Neimenggu Wenwu Kaogu (2):8-15 and 30.

Liu, G.X., et al.

2004 Report of the 2002-2003 season excavation of the Xinglonggou site in Chifeng City, Inner Mongolia (in Chinese with English abstract). Kaogu (Archaeology) (7):31-39.

Liu, X., Jones, M. K., Zhao, Z., Liu, G., & O'Connell, T. C.

2012). The earliest evidence of millet as a staple crop: New light on neolithic foodways in North China. *American Journal of Physical Anthropology*, *149*(2), 283-290.

Liu XY, Zhao ZJ, Liu GX

2015 Xinglonggou. In: Barker G, Goucher C (eds) The Cambridge World History, vol 2. A world with agriculture, 12,000 BCE-500 CE. Cambridge University Press, Cambridge, pp 335–352

Lu H, Zhang J, Liu K-B et al.

2009 Earliest domestication of common millet (*Panicum miliaceum*) in East Asia extended to 10,000 years ago. Proc Natl Acad Sci 106:7367–7372

Lu, Tracey L. D. 1999. *The Transition from Foraging to Farming and the Origin of Agriculture in China*. BAR International Series 774. Oxford: British Archaeological Reports.

Luo, Y.B.

2012 Domestication, Husbandry and Ritual Usuage of Pigs in Ancient China. Beijing: Science Press.

Ma XJ, Jing YZ, Jia XB, Zhao ZJ.

2010 Flotation results and analysis of the plant remains from the Wujiacun Site in 2010 (in Chinese). *Dongfang Kaogu (Oriental Archaeology)* 11(1): 387-393.

Pechenkina, E. A., Ambrose, S. H., Xiaolin, M., & Benfer Jr, R. A.

2005. Reconstructing northern Chinese Neolithic subsistence practices by isotopic analysis. *Journal of archaeological Science*, *32*(8), 1176-1189

Qin, L, Fuller, D Q

2009. Appendix 3. The Nanjiaokou site 2007 excavated Early to Mid Yangshao plant remains. In: Henan Provincial Institute of Cultural Relics and Archaeology (ed.) Nanjiaokou Site in Sanmenxia. Science Press, Beijing. Pp. 427-435 [in Chinese]

Ren, SN.

1996 Several major achievements in Early Neolithic China, ca. 5000 BC. Trans. by Wing Kam Cheung. Kaogu 1996: 37‐49.

Shelach-Lavi, G., Teng, M., Goldsmith, Y., Wachtel, I., Stevens, C.J., Marder, O., Wan, X., Wu, X., Tu, D., Shavit, R. and Polissar, P.

2019. Sedentism and plant cultivation in northeast China emerged during affluent conditions. PloS one, 14(7), p.e0218751.

Song,J.X.

2011. The Agricultural Economy during the Longshan Aeriod: An Archaeobotanical Perspective from Shandong and Shanxi. PhD Dissertation, University College London.

Song, Y.B.

2012 Zooarchaeological Research of the Neolithic Haidai Region, Shandong University.

—

2016 Zooarchaeological study of faunal remains from Houli culture sites in the Jinan region (in Chinese with English abstract). Huaxia Archaeology (3):53-59.

Stevens, C.J., Shelach-Lavi, G., Zhang, H., Teng, M. and Fuller, D.Q.

2021 A model for the domestication of Panicum miliaceum (common, proso or broomcorn millet) in China. *Vegetation History and Archaeobotany*, *30*(1), pp.21-33.

Sun B, Wagner M, Zhao ZJ et al.

2014 Archaeological discovery and research at Bianbiandong early Neolithic cave site, Shandong, China. Quaternary International 348:169–182

Sun YG, Zhao ZJ and Ji P.

2016. Study on the remains of plants unearthed from the site of the prehistoric settlement of Hamin. Haxia Archaeology , 2016(2): 45-52.

Wu, W., Wang, X., Wu, X., Jin, G., & Tarasov, P. E.

2014. The early Holocene archaeobotanical record from the Zhangmatun site situated at the northern edge of the Shandong Highlands, China. Quaternary International, 348, 183-193.

Wang, Jihua.

1984 Charred foxtail millets found in the Shawoli site, Xinzheng. *Nongye Kaogu* *[Agricultural Archaeology]* 1984(2): 194–207. [In Chinese.]

Wang X, Shang X, Jiang HE et al.

2015 Preliminary analysis of flotation results from two sites in the Baishui River Basin, Shaanxi (in Chinese). Archaeology and Cultural Relics]2015(2):100-104

Wang YQ, Zhang P, Jin GY

2011 The results and analysis of plant flotation in 2007 at the Gouwan Site in Xixhuan, Henan (in Chinese). Sichuan Cultural Relics 2011(2): 80-92

Yan W (1992) Origins of agriculture and animal husbandry in China. In: Aikens CM and Song NR (eds) Pacific Northeast Asia in Prehistory: hunter-fisher-gatherers, farmers, and sociopolitical elites. Pullman. Washington: Washington State University Press, pp. 113-123.

Yang XY, Wan Z, Perry L et al.

2012 Early millet use in northern China. Proc Natl Acad Sci 109:3726–3730

Yang XY, Ma ZK, Li J, Yu JC, Stevens C, Zhuang YJ

2015 Comparing subsistence strategies in different landscapes of North China 10,000 years ago. The Holocene 25:1957–1964

Zhang, Chi and Hsiao‐chun Hung.

2013. Jiahu 1: earliest farmers beyond the Yangtze River. *Antiquity* 87(33): 46‐63

Zhang JN, Xia ZK, Zhang XH

201) Research on charred plant remains from the Neolithic to the Bronze Age in the Luoyang Basin (in Chinese). China Science Bulletin 59 (34): 3388-3397

Zhang, W., M. Han, and Y. Li

2003 The causes of disappearance of ancient lakes in south coase plain of Laizhou Bay, Shandong province (in Chinese with English abstract). Journal of Palaeogeography 5(2):224-231.

.

Zhao, M.C.

1990 Seasonality of precipitation on the Loess Plateau and crop water stress (in Chinese with English abstract). Journal of Natural Resources 5(3):218-229.

Zhao, Zhijun, Zhao, Chaohong Yu, Jincheng, Wang, Tao, Cui, Tianxing, Gu, Jingning,

2020. Archaeobotanical results from the Donghulin site of Beijing and an analysis (in Chinese). *Kaogu* 2020 (7):99-106.

Zhao ZJ.

2017 The development of agricultural production and the establishment of agricultural society in the Yangshao culture period: Analysis of the flotation results from the Yuhuazhai site (in Chinese). Jianghan Kaogu 153(6):98-108

Zuo, Xinxin, Houyuan Lu, Jianping Zhang, Can Wang, Guoping Sun and Yunfei Zheng.

2016. Radiocarbon dating of prehistoric phytoliths: a preliminary study of archaeological sites in China. *Scientific Reports* 6: 26769
